# Proceedings from the Second Annual Conference of the Norwegian Network for Implementation Research

**DOI:** 10.1007/s43477-022-00069-w

**Published:** 2022-11-23

**Authors:** Karina M. Egeland, Thomas Engell, Jeanette Halvorsen, Cecilie Varsi

**Affiliations:** 1 Norwegian Centre for Violence and Traumatic Stress Studies (NKVTS), Oslo, Norway; 2 Centre for Child and Adolescent Mental Health, Eastern and Southern Norway, Oslo, Norway; 3 Norwegian Centre for Learning Environment and Behavioral Research in Education, Faculty of Arts and Education, University of Stavanger, Stavanger, Norway; 4 Faculty of Health and Social Sciences, University of South-Eastern Norway, Drammen, Norway

**Keywords:** Conference proceedings, Implementation research, Research network, Norway

## Abstract

In 2019, Norwegian implementation researchers formed a network to promote implementation research and practice in the Norwegian context. On November 19th, 2021, the second annual Norwegian implementation conference was held in Oslo. Ninety participants from all regions of the country gathered to showcase the frontiers of Norwegian implementation research. The conference also hosted a panel discussion about critical next steps for implementation science in Norway. The conference included 17 presentations from diverse disciplines within health and welfare services, including schools. The themes presented included stakeholder engagement, implementation mechanisms, evaluations of the implementation of specific interventions, the use of implementation guidelines and frameworks, the development and validation of implementation measurements, and barriers and facilitators for implementation. The panel discussion highlighted several critical challenges with the implementation of evidence-informed practices in Norway, including limited implementation competence and capacity among practice leaders and workforces, few opportunities for education in implementation science, limited implementation research in the Norwegian context, scarce funding possibilities for implementation research, and a lack of long-term perspectives on implementation processes. Overall, the 2021 Norwegian implementation conference showed an encouraging sign of a maturing field of science in Norway. The more voluminous proceedings from the 2020 conference called for several important advancements to improve implementation science and practice in Norway, and the 2021 conference indicates that steps have already been taken in favorable directions in terms of, for instance, research designs and measurements. However, there are still unexploited potentials for improvements in implementation research, funding, policies, and practice. Norwegian implementation researcher should be mindful of the challenges and potential pitfalls implementation science currently face as a scientific discipline.

**Supplementary Information:**

The online version contains supplementary material available at 10.1007/s43477-022-00069-w.

The Norwegian Network for Implementation Research (NIMP) was launched in 2020 to connect Norwegian researchers, policymakers, practitioners, and others interested in implementation science and share knowledge from implementation research among its members (Engell et al., 2021) (see Table 1 for key terms and definitions used in the article). In 2021, NIMP opened membership registration and established a group on Facebook for information sharing. The number of members has risen steadily since its establishment, and NIMP has 252 registered members and 519 followers on Facebook as of October 2022. In line with its goals, NIMP started organizing annual implementation conferences to present contemporary implementation research conducted in Norway. The first conference in 2020 brought together 144 participants online. Common themes from the studies presented at the conference were implementation barriers and facilitators, experiences with different implementation strategies, and implementation process evaluations—many from ongoing studies (Engell et al., 2021). The conference presentations and the number of attendees were encouraging signs of a proliferation of implementation research and interest in Norway. However, the conference also raised awareness of the need to increase the spread of knowledge from implementation science to research, policy, and practice across disciplines and sectors with health and welfare (including schools).

**Table 1:** Key terms and definitions

Term	Definition
Implementation	The act of carrying intentions into effect (Peters et al., 2013) In this article, *intentions* refer to evidence-informed practices, programs, policies, guidelines, systems, or other improvement efforts (collectively referred to as interventions)
Implementation research	Scientific inquiry into questions concerning implementation—the act of carrying intentions into effect
Implementation science	The scientific discipline of implementation research
Research network	Organized structure of ties among individuals with mutual interests in a scientific discipline or topic (Nohria & Eccles, 2000)

The second conference was held in-person on November 19th, 2021, and brought together 90 participants from all regions of the country. The conference aimed to bring together NIMP members and others interested in implementation science and practice, showcase the frontiers of Norwegian implementation research, and stimulate debate about critical next steps for implementation science in Norway. The content of the conference, to a large extent, represents the state of implementation research in Norway, and with these proceedings, we aim to:

1. Showcase contemporary implementation research from Norway, and
2. Provide insight into current debates about how to improve the implementation of evidence-informed practices in Norwegian health and welfare systems (including schools and kindergartens).

## The Second Annual NIMP Conference

The organizing committee consisted of the elected NIMP board members, five representing research institutions or universities conducting implementation research, and one representing a community welfare service dealing with implementation practice. Of the 90 attendees at the conference, 67 were women. Twenty-six were affiliated with research and quality improvement organizations, 16 with academia, 14 with policy, 13 with municipalities, 12 with private organizations or foundations, and nine with hospitals.

Abstracts were sought for oral and poster presentations from research communities in the health and welfare sectors through emails and social media postings. The invitation specified that the main focus of the conference was on the implementation of evidence-informed practices (including interventions, quality improvements, guidelines etc.). Therefore, contributions primarily focused on the development or effectiveness of a particular practice would be deprioritized. The invitation also provided examples of relevant topics for presentations, including (but not limited to) theory and methods within implementation research, fidelity and adaptation in implementation, evaluation of implementation strategies and processes, barriers and facilitators for implementation, sustainability, scaling up/out, evaluation of context and capacity for implementation, and policy implementation—all related to a Norwegian context. The conference was hosted in person in Oslo, without the opportunity to attend remotely via stream. The program consisted of one keynote talk, oral presentations, a poster session, and a panel discussion.

## Methods

### Data Collection

We collected data from oral and poster presentations from submitted abstracts and PowerPoint presentations used in the talks, after the presenters had given their consent. The abstracts were collected in the call for abstract submission procedure. The PowerPoint slides were gathered prior to or during the conference by the organizing committee. We collected data from the panel discussion by using observation and taking notes. Demographics of conference attendees were collected from the conference registration form.

### Data Analysis

We conducted content analysis in order to summarize the content of the oral and poster presentations at the conference. Mainly, the abstracts formed the basis for the analysis. If something was unclear, we used the PowerPoint slides. Two of the authors (CV and KME) carried out the analysis, using an Excel spreadsheet to organize text into the following codes: Name, Affiliation, Title, Aim, Theme, Context, Study Design, Methods and Implementation Framework. All authors were involved in the final stage of the analysis to reach agreement of the analysis. All who submitted abstracts were presented with the analysis and approved the content by e-mail. The initial summary of the panel discussion was written by TE informed by notes, and all authors edited and agreed on the final summary jointly.

### Summary of Presentations

Seventeen abstracts were submitted for the conference. Three of the organizing committee members reviewed all the abstracts. The conference consisted of 18 presentations, of which nine were oral presentations of accepted abstract submissions, eight were poster presentations of accepted submissions, and one invited keynote presentation (#1 in Table 2). Table 2 depicts the title of the oral presentations and the name of the presenters. Table 3 depicts the titles of the poster presentations and the name of the presenters. The summary does not distinguish between oral and poster presentations. However, each presentation is referenced with a hashtag and number corresponding to numbers in Tables 2 and 3, distinguishing between oral and poster formats. Abstracts in Norwegian and English are available in supplementary files 1 and 2.

**Table 2:** Presenters and oral presentations at the 2021 NIMP conference

Presenters	Title of presentation	#
Thøgersen, D. G., Lønnum, K	The responsibility of authorities, researchers, and practitioners for succeeding in implementation	1
Meshkovska, B., Forberger, S., Scheller, D. A., Wendt, J., Castellari, E., Tiboldo, G., Luszczynska, A., Lien, N	Barriers and facilitators to implementation of the EU School Fruit and Vegetables Scheme: cross-country study using the Consolidated Framework for Implementation Research (CFIR)	2
Halvorsen, J	Development and testing of a web application utilized for evaluation of fidelity in implementation and process evaluation (IPE)	3
Graverholt, B., Espehaug, B, Potrebny, T., Igland, J., Ciliska, D	The IMPAKT intervention in nursing homes—development, implementation, and evaluation of a complex intervention to meet relevant needs	4
Potrebny, T., Graverholt, B., Espehaug, B., Igland, J., Ciliska, D	Organizational context in Norwegian nursing homes: a cross-sectional study	5
Nygaard, E., Edvolll, M., Havighurst, S. S	Implementing Tuning in to Kids in Norwegian kindergartens: A study of implementation processes in a group-randomized study	6
Halvorsen, M. J., Henderson, C., Hågå, M. G., Bergseth, K., Byhring, M., Eggen, T. L. B., Gustavsen, H., Rosseland, I., Nordvik, J. E., Hornby, T. G., Moore, J. L	Sustainable implementation of high-intensity gait training for patients in rehabilitation after stroke	7
Bø, E., Moore, J. L., Erichsen, A., Rosseland, I., Halvorsen, M. J., Bratlie, H., Hornby, T. G., Nordvik, J. E.	Development and results of a successful implementation plan for high-intensity gait training for patients after stroke	8
Egeland, K., Skar, A-M. S.	Study of Leadership and Organizational Change for Implementation (LOCI) as a strategy for implementing knowledge-based practice in mental health care	9
Mbalilaki, J. A., Moore, J., Graham, I.	Systematic overview of knowledge translation in rehabilitation research	10

Titles translated from Norwegian into English by the authors, and approved by the presenters

**Table 3:** Presenters and poster presentations at the 2021 NIMP conference

Presenters	Title of presentation	#
Fredriksen, T	Implementation of an RCT in the specialist health service: Challenges and opportunities	11
Steinskog, T. L., Tranvåg, O., Nortvedt, M., Ciliska, D., Graverholt, B	Implementing an integrated knowledge translation intervention in nursing homes: experiences of practice development nurses	12
Braathu, N., Borge, R. H., Endsjø, M., Peters, N	Validations of the Implementation Leadership Scale, Implementation Climate Scale, Implementation Citizenship Behaviour Scale and the AIM, IAM and FIM scales in a Norwegian mental health care setting	13
Rimehaug, S. A	Brief introduction to the KTA Knowledge to Action framework	14
Haukeland, Y. B., Vatne, T. M., Kongshavn, A-H., Nes, R. B	Siblings of children with chronic disorders: A survey of preventive mental health work in Norwegian municipalities and prospective acceptance of the group-based measure SIBS	15
Graverholt, B., Strømme, H., Ciliska, D	A guide for knowledge translation and implementation—in Norwegian!	16
Kvæl, L. A. H	The IPIC study: The effect of an interprofessional learning program on user participation among older people in short-term rehabilitation: A quasi-experimental study	17
Igland, J., Potrebny, T., Bendixen, B. E., Haugstvedt, A., Espehaug, B., Titlestad, K. B., & Graverholt, B	Translation and validation of the Alberta Context Tool for use in Norwegian nursing homes	18

Titles translated from Norwegian into English by the authors and approved by the presenters

### Themes

The keynote presentation addressed stakeholder responsibilities for successful implementation. The speakers emphasized the importance of dialogue and collaboration between the national authorities, practice contexts delivering services, and research communities (e.g., policy, community, and academic partnerships). Moreover, the presentation demonstrated differences between top-down and bottom-up initiatives, and that bottom-up initiatives can experience more system-level barriers, and be more difficult receiving governmental support and research fundings [#1].

Five of the presentations were from one large study implementing a complex intervention addressing clinical quality improvements and competence in nursing homes (i.e., the IMPACT study) [#4]. These presentations included translation, cross-cultural adaptation, and validation of the Alberta Context Tool (Estabrooks et al., 2009) to Norwegian [#18], organizational context assessment [#5], experiences from conducting implementation [#12], and the translation of a guiding implementation framework to Norwegian [#16]. Two more presentations addressed aspects of frameworks, of which one presented an introduction to the Knowledge-to-Action framework (Graham et al., 2006) [#14], and the other presented a systematic overview of the same framework [#10].

One presented validations of several implementation measures for use in the Norwegian contexts [i.e., Implementation Leadership Scale (Aarons et al., 2014)]. Implementation Climate Scale (Ehrhart et al., 2014), Implementation Citizenship Behaviour Scale (Ehrhart et al., 2015), and Acceptability of Intervention Measure, Intervention Appropriateness Measure, and Feasibility of Intervention Measure (Weiner et al., 2017) [#13]. Another presentation addressed the development of a fidelity assessment tool [#3], and four presentations reported on the evaluation of implementation outcomes [#6, #8, #9, #17], one of the presentations focused on testing implementation mechanisms [#6]. Finally, one presented barriers and facilitators for implementation [#2], one reported on the prospective acceptability of an intervention [#15], and one addressed the implementation sustainability of an intervention [#7]. One presentation did not address aspects related to implementation research specifically [#11].

### Contexts

Five presentations addressed implementation studies conducted in nursing homes [#4, #5, #12, #16, #18], four in the context of rehabilitation [#7, #8, #10, #17], and four in child and adult mental health services [#9, #11, #13, #15]. One presentation was from an implementation study in kindergartens [#6], one from middle school [#3], and one from policy implementation in elementary schools [#2]. Two presentations were not related to a specific implementation context [#1, #14].

### Theories and Frameworks

Ten of the presentations addressing implementation studies stated that they had used implementation theories, models, and/or frameworks. Four studies used the Knowledge-To-Action framework (KTA, Graham et al., 2006) [#7, #8, #10, #14]. One addressed KTA in a general presentation of its content [#14], and the other was a systematic review of its use for implementation in the context of rehabilitation [#10]. The other two studies combined the use of the KTA with the NHS Sustainability Model (Maher et al., 2010) [# 7] and the Consolidated Framework for Implementation Research (CFIR) (Damschroder et al., 2009) to guide and evaluate implementation [#8]. Another study combined the use of CFIR with the Implementation Outcome Framework (Proctor et al., 2011) [#6], while two studies used the CFIR exclusively [#2, #15]. Two presentations stated that they used the Integrated Knowledge Translation framework (Jull et al., 2017). [#4, #12], one of them in combination with the Medical Research Council guidance for process evaluation of complex interventions (Moore et al., 2015) [#4]. Finally, one of the studies used the Exploration, Preparation, Implementation, Sustainment framework (EPIS) (Aarons et al., 2011) [#9].

### Study Designs and Methods

Of those that presented original empirical studies, the study designs used were quantitative [#9, #13], qualitative [#2, #7, #12] and mixed methods [#3, #4, #6, #8, #15]. There were several presentations from studies with a randomized controlled design [#4, #6, #9, #13], but only two studies reported results based on a comparison between randomized groups [#6, #9].

## Summary of the Panel Discussion

One of the keynote features of the program was a panel discussion with the overarching theme: “Effective interventions are not being implemented—what can we do about it?” The panel consisted of four debaters and one moderator, including the spectators in the audience. Debate participants are depicted in Table 4.

**Table 4:** Participants at the panel debate

Participant	Occupation	Affiliation
1	Special advisor	Hospital
2	Department director	Research and innovation funder
3	Research leader	National research centre
4	Associate professor	Regional research centre
Moderator	Vice-dean	University

The moderator introduced the debate by emphasizing that many evidence-informed practices, treatments, interventions, and programs in Norway are used to a limited extent in practice within health, education, and other welfare services. As a result, clients, patients, or students often receive random, outdated, and/or suboptimal services. The moderator then asked the participants to reflect upon how stakeholders such as researchers, bureaucrats and practitioners together can strengthen the implementation of evidence-informed practices.

The debate had a fluctuating start, with the participants speaking their core issues and arguments with limited overlap. In the initial round of the debate, participants mainly presented general arguments drawn from experiences in their respective contexts. One debater remarked that rapid and successful implementation is possible in times of urgency, as demonstrated during the COVID 19 pandemic. However, the debater had also observed limited implementation competence and frequent implementation fatigue in real-world practice. A couple of the debaters stressed the importance of implementation training in the education of leaders and practitioners and argued for bringing implementation science and practice into the education systems. Panelists endorsed these remarks, and there was agreement about the need to ensure implementation competencies across workforces within the health, welfare, and education sectors.

One debater stressed the need for more implementation research, and noted observations of technical language use in implementation science being a barrier to implementation practice. Another debater emphasized the importance of motivated groups and individuals for successful implementation, and that implementation works best when users are involved from the beginning of the implementation process, especially when the need for implementation is expressed from the bottom-up. It was also noted that traditional platforms for disseminating evidence about interventions and other practices might need to find better ways of providing implementation support and resources for their evidence syntheses to have more impact in practice. Lastly, networking through initiatives such as NIMP was mentioned as an activity that can help bridge implementation research and practice, strengthen the Norwegian implementation community, and spread awareness and knowledge about implementation science to policymakers, funders, researchers, and practitioners.

When spectators were allowed to ask questions, the debate shifted towards funding for implementation studies and the lack of dedicated research funds for implementation science. One participant emphasized that research funders such as The Research Council of Norway (i.e., NFR; the Norwegian government's funder of research) are dedicated to implementation in that receiving grants require well-articulated and realistic implementation plans. The participant also mentioned that NFR is now common to request evaluation of the implementation process alongside research on evidence-informed interventions in several of their calls for funding. As a response, another participant stressed that the emphasis on evidence-informed interventions is all fine and well, and we have numerous interventions ready for implementation. However, the participant stressed that we are far from evidence-informed implementation in Norway because we lack evidence about effective dissemination and implementation. Subsequently, emphasis was placed on the critical need for more dedicated implementation research and that process evaluations playing second fiddle to intervention research is insufficient. It was also noted that process evaluations and studying barriers and facilitators can be useful, but substantial progress requires studies and designs tailored to ask questions about implementation first.

Furthermore, another participant from the audience argued that the health and welfare sectors, especially policymakers and funders, have an inappropriately narrow view of the implementation process as a short, time-limited concept. For example, the participant mentioned that politicians typically need initiatives to be implemented and evaluated before a new election (maximum four years in Norway) and that research funders' have a default study template of 3–4 years. As an analogy of how implementation processes should be conceptualized, the participant described the city of Amsterdam “floating” on wooden piles in continuous need of oversight, maintenance, and replacement to keep the city above sea level, and the participant stressed that only well-structured systems with long-term perspectives could sustain and normalize such a process over time. Another participant from the audience noted that it was uplifting to hear such perspectives in presentations during the day and added that if we get policymakers and funders to adopt similar perspectives, we would be well-equipped to make substantial progress with implementation in Norway. Agreement between the panel and the audience was notable.

## Discussion

The panel discussion at the second annual NIMP conference about how to increase the implementation of evidence-informed practices outlined critical needs for improvement in three major intertwined areas: (1) Implementation competence and capacity in practice settings, (2) investment in implementation science and practice, and (3) dedicated implementation research in Norwegian contexts. In debating building implementation capacity in natural practice settings, the panel suggested several strategies that resonate with the implementation literature such as community-academic partnerships, formal education of leaders, staff, and students on how to practice and succeed with implementation, as well as more informal training opportunities in implementation leadership and practice (Juckett et al., 2022). Related to capacity building, the panel and audience call for more active spread of implementation science to policymakers and funders to raise awareness of the scale and longevity needed to make substantial progress in implementation research and practice. Studies and reports also indicate that policymakers limitedly use implementation science in forming and implementing policy (Cervantes et al., 2021; Strehlenert et al., 2015). Therefore, reaching policymakers with updated knowledge about implementation science may influence policy priorities and, in turn, facilitate improved conditions for dedicated implementation research and implementation capacity building across Norwegian health and welfare settings.

The presentations at the second annual NIMP conference included themes often present at implementation conferences, and that commonly occur in contemporary implementation studies internationally, such as stakeholder engagement (Triplett et al., 2022), evaluations of the implementation of specific interventions (Strehlenert et al., 2015), the use of implementation guidelines and frameworks (Albrecht et al., 2022), and barriers and facilitators for implementation (Chen et al., 2022), such as organizational and contextual determinants. Fewer presentations than in the 2020 NIMP conference focused on barriers and facilitators for implementing interventions (Engell et al., 2021) and more on the multi-level strategies and mechanisms that drive effective implementation. In addition, developing and validating measurements of implementation determinants and outcomes appears to be a priority for Norwegian implementation researchers. Similarly, a few presentations also addressed cultural adaptation of implementation frameworks and measurements, which is important to ensure fit with Norwegian contexts. These developments are encouraging and in line with calls for advancements expressed in the proceedings from the 2020 conference (Engell et al., 2021). However, we urge Norwegian implementation research to take note of potential pitfalls implementation science, in general, is at risk of encountering, such as limitations in the practicality and applicability of implementation frameworks and strategies for natural practice settings and underestimating structural and contextual variation (Beidas et al., 2022).

Presentations were from diverse disciplines such as health care and mental health services, education, social care, and other welfare services. Target populations of innovations being implemented ranged from the age of children in kindergarten to the elderly in nursing homes. Several studies presented the use of well-known implementation models and frameworks. The studies represented both qualitative and quantitative methods, with an overweight of studies using combined methods and some purposefully using mixed methods. The combination of the commonalities between the presentations mentioned above and the breadth of themes, frameworks, and settings indicate that Norwegian implementation research appears to be growing in various directions with some common meta about critical research needs (e.g., implementation strategies, mechanisms, and measurement across contexts). This can be perceived as progress toward generalizable implementation science in the Norwegian context.

Although few studies fully utilized a randomized controlled design to answer implementation questions, there were nevertheless several studies derived from ambitious, randomized designs, which may be a positive sign of large implementation studies now being prioritized in Norway. These studies may present valuable contributions to the implementation knowledge base in the coming years. While we applaud this development and strongly encourage the use of randomized controlled trials (RCT) when appropriate, it is also important to note that RCTs are not necessarily the gold standard designs for answering questions about implementation (Brownson et al., 2022; Minary et al., 2019). We warn against relying too heavily on traditional perspectives on what constitutes valid and useful scientific evidence for implementation (Brownson et al., 2022). Instead, we encourage awareness and recognition of diverse perspectives on theory, evidence, and implementation as a concept, and that the questions asked about implementation inform the selection of designs, methods, and processes, and not the other way around.

Norway is a sparsely populated country with extensive social and economic capital Such conditions should be favorable for implementation research and practice. However, implementation science is still underutilized in Norwegian practice, policy, and research (Engell et al., 2021). NIMP growing fast as a network may coincide with increasing awareness of the importance of implementation science for health and welfare improvements. Regardless of whether or not NIMP contributed to the increase in awareness, NIMP can use this momentum to help further increase interest in implementation science and practice exponentially within the health and welfare systems. Continued growth in NIMP may therefore support growth in the field.

Several take-home messages from the conference regards implementation science in practice (i.e., implementation capacity-building in practice, dissemination of implementation science in education, policy and practice, and community-academic partnerships). Hence, a natural development for the next NIMP conference is to also call for presentations of implementation efforts in natural practice settings, which means experiences and lessons from using implementation science in practice without necessarily being implementation research.

## Conclusion

The second annual NIMP conference in Norway showcased a wide array of implementation research within Norwegian health and welfare settings. The research studies and results presented can help move implementation science and practice forward—in Norway especially, and also internationally. In the continued progression of implementation science, we urge researchers to keep being mindful of the practice-based nature of implementation and the implications that nature may have for scientific theories, designs and methods. A panel discussion highlighted several potential improvements that may help the implementation of evidence-informed practices, such as building implementation competence and capacity in practice settings, influencing policy priorities by spreading knowledge and awareness of implementation science, and more heavy investments in dedicated implementation research.

## Supplementary Information

Below is the link to the electronic supplementary material.

Supplementary file1 (DOCX 48 kb)

Supplementary file2 (DOCX 71 kb)

## Data Availability

The data generated during the events presented in this article are not publicly available due to national ethical regulations, but may be available from the corresponding author on reasonable request, if approved by the owners of the data.
