# Supplementary material for: Proceedings from the Second Annual Conference of the Norwegian Network for Implementation Research
Source: Glob Implement Res Appl. 2022 Nov 23;2(4):332–9. doi: 10.1007/s43477-022-00069-w (PMC9684746; doi:10.1007/s43477-022-00069-w)
Supplement: Supplementary file 1 — Supplementary file1 (DOCX 48 kb) [file 43477_2022_69_MOESM1_ESM.docx]

## ABSTRACTS IN NORWEGIAN

The NIMP conference

November 19, 2021

Oslo, Norway

## #2 Barrierer og fasilitatorer for implementeringen av EU sin skolefrukt- og grønnsaksordning: tverrnasjonal studie som benytter Consolidated Framework for Implementation Research (CFIR)

### Meshkovska, B., Forberger, S., Scheller, D. A., Wendt, J., Castellari, E., Tiboldo, G., Luszczynska, A., Lien, N. Department of Nutrition, Universitetet i Oslo

**Bakgrunn:** Siden 2009/2010 har EUs skolefrukt- og grønnsaksordning vært implementert i store deler av EU. Den har sin opprinnelse EUs felles landbrukspolitikken og har tre hovedhandlinger: levering av frukt og grønnsaker til barn i skolen, medfølgende pedagogiske tiltak og informasjon.

**Mål:** Målet med denne studien er å identifisere barrierer og fasilitatorer for implementering av ordningen basert på oppfatninger fra de ansvarlige på myndighetsnivå og vurdere anvendeligheten av the Consolidated Framework for Implementation Research for dette formålet.

**Metode**: Tjuetre semistrukturerte intervjuer (N=29) ble gjennomført med personer fra landbruks-, helse- og utdanningsdepartementer, på tvers av 10 EU-medlemsland og med en representant fra EU-nivået. Kvalitative data ble opprinnelig kodet induktivt, og deretter ble den induktive kodingen kartlagt til domenene/konstruksjonene/subkonstruksjonene til CFIR. Det nasjonale nivået ble ansett som den indre rammen for CFIR. Barrierer og fasilitatorer ble deretter identifisert innenfor hver konstruksjon/subkonstruksjoner.

**Resultater:** Følgende CFIR-konstruksjoner (og barrierer og fasilitatorer knyttet til hver) ble funnet relevante: 1) Ordningens karakteristikker: 'ordningens kilder', 'bevisstyrke og kvalitet', 'relativ fordel', 'adaptabilitet', 'designkvalitet og emballasje' og 'kostnad'; 2) ytre setting: 'kosmopolitisme', 'gruppepress' og 'ekstern politikk og insentiver'; 3) indre setting: 'strukturelle karakteristikker', 'nettverk og kommunikasjon', 'implementeringsklima' og 'hvor klar man er for implementering'; 4) karakteristikker ved individer: 'kunnskap og tro om intervensjonen'; 5) prosess: 'engasjement', 'utføre' og 'reflektere og evaluere'.

**Konklusjon:** CFIR er egnet for bruk på nasjonalt nivå, for å identifisere barrierer og fasilitatorer for policyimplementering.

## #3 Refleksjoner omkring utvikling og testing av app til bruk for måling av fidelitet i implementering og prosessevaluering (IPE)

### Halvorsen, J. Læringsmiljøsenteret, Universitetet i Stavanger

Mitt PhD-prosjekt er en del av RCT-studien Resilient, hvor målet er å bedre studentenes velvære og motivasjon, redusere emosjonell stress og ensomhet, og bedre akademisk utfall. Min avhandling har tentative tittelen: «Evaluere fidelitet og dose i Resilient-intervensjonen», hvor intervensjonen består av sosiale og emosjonelle læringsmål (SEL) fordelt på 25 timer i følgende fem hovedkomponenter: sosiale relasjonsferdigheter, mindfulness, problemløsning, emosjonsregulering og vekst i tankesett. Et hovedfokus i avhandlingen er evaluering av fidelitet (dvs. om intervensjonen er implementert som planlagt) ved å bruke en webbasert fidelitetsapplikasjon (referert til som appen), utviklet som et verktøy for dette målet. Appen er benyttet av implementørene (lærerne) for å rapportere gjennomføringen av timene når de implementerer målene i klasserommene.

I denne presentasjonen vil fokuset være på følgende forskningsspørsmål: Hvordan måler lærerne bruken av den webbaserte fidelitetsappen? Målet er å evaluere bruken av appen, å forstå hvis, og om mulig hvordan appen kan benyttes som et verktøy også i andre prosjekter. Dette er spesielt interessant knyttet til prosjekter hvor det er nødvendig å måle fidelitet og implementeringskvalitet i implementering og prosessevaluering. PhD-prosjektet benytter blandet metode og er kvalitativt drevet, hvor datainnsamlingen gjennomføres med fokusgruppeintervjuer, individuelle intervjuer, observasjon og datainnsamling via appen. Data fra et pilotprosjekt vil presenteres her. Data er samlet inn fra et digitalt fokusgruppeintervju (zoom) med 7 lærere som piloterte appen deres klasserom før implementeringen startet opp. Lærerne, som ikke tar del I intervensjonen, er rekruttert fra hele Norge. Appen er inspirert av fidelitysjekklisten og det teoretiske rammeverket som Resilient-intervensjonen bygger på. Lærerne testet ut en del av timene fra intervensjonen (5 timer: 1 time fra hver av komponentene). Lærerne registrerte deretter i hvilken grad timene ble gjennomført som planlagt, noe som krevde erfaring med å teste ut appen før de deltok i fokusgruppeintervjuet. Hovedmålet for intervjuet var å samle inn informasjon fra lærernes erfaringer med å bruke appen.

Preliminære analyser viser at appen kan være nyttig verktøy både for lærerne og forskere knyttet til slike intervensjoner. Preliminære resultater også viser potensiale for forbedringer i appen, slik som et ønske om tydeligere instruksjoner før bruk, samt behov for felt hvor man kan legge inn kommentarer. Kunnskapen og innsikten fra lærerne har bidratt til viktige perspektiver fra praksisfeltet om bruk av app som måler fidelitet. Appen kan være et nyttig verktøy for å måle kvalitet og forbedre kvalitet av IPE. PhD-prosjektet har potensiale for å bidra til å lukke kunnskapsgapet relatert til å evaluere fidelitet av IPE, på tvers av relevante forskningsområder og forskningsfelt.

## #4 IMPAKT intervensjonen i sykehjem – utvikling, implementering og evaluering av en kompleks intervensjon for å møte relevante behov

### Graverholt, B., Espehaug, B, Potrebny, T., Igland, J., Ciliska, D. Høgskulen på Vestlandet

**Bakgrunn:** Sykehjem som helsetjenestesetting og arbeidssted blir stadig mer kompleks i tråd med nye oppgaver og skjerpete krav. Dette krever at profesjoner i sykehjem må utvikle praksis og kompetanse i høyt tempo. Men vi vet lite om hvilke utfordringer denne sektoren har for å sikre at utviklingen skal være kunnskapsbasert. Hensikt Å utvikle og implementere en intervensjon som svarer på kliniske spørsmål og kompetansebehov i kunnskapsbasert praksis i sykehjem.

**Metode:** Metodiske rammeverk som ligger til grunn for studien er MRC framework of complex interventions og Integrated Knowledge Translation. For å utvikle intervensjonen kartla vi kliniske kunnskapshull og kompetansebehov i sykehjem, gjennom kvalitative og kvantitative metoder. Vi utforsket rollefordeling og ansvar for kunnskapsbasert praksis gjennom intervjuer med ledere på ulike nivå. Behov rettet mot det å jobbe kunnnskapsbasert ble kartlagt

**Resultat:** Vi utviklet en klyngerandomisert kontrollert studie blant 19 sykehjem, informert av behov ytret fra sektoren selv. Gjennom ulike delstudier informerte vi viktige beslutninger for intervensjonen: Ett klinisk viktig område med behov for forbedring, deltakere, kontekstuelle hensyn og aktive komponenter.

**Konklusjon:** En kunnskapsbasert utvikling av tjenestene krever at hele organisasjonen harmoniseres, med avklarte roller og rett kompetanse. Det tette samarbeidet mellom forskere fra HVL og sykehjem som helsetjenestesetting i IMPAKT har ført til flere gjensidige fordeler. Disse inkluderer relevant forskning informert av de som jobber der, og enklere og bedre rekruttering av deltakere til studier.

## #5 Organisasjons kontekst I Norske sykehjem: Ett tverrsnitt studie.

### Thomas Potrebny, Donna Ciliska, Birgitte Espehaug, Jannicke Igland & Birgitte Graverholt, Høgskulen på Vestlandet.

**Bakgrunn:** Gunstig helseorganisasjonskontekst (dvs.: arbeidsmiljøet) er assosiert med bedre pasientresultater og økt arbeidstilfredshet. Langtidspleie anses ofte som et utfordrende arbeidsmiljø preget av høye jobbkrav, lav jobbkontroll, høyt arbeidstempo og regelmessig eksponering for trusler og vold som kan påvirke pasientbehandlingen og øke personalutskiftingen. Denne studien sitt mål er å utforske trekk ved en gunstig organisatorisk kontekst i norske sykehjem.

**Metoder:** Denne studien er en tverrsnittsstudie av registrerte sykepleiere og praktiserende sykepleiere med lisens i Bergen, Norge (n = 1014). Organisasjonskontekst ble målt med Alberta Context Tool. K-betyr klyngealgoritme ble brukt for å skille mellom gunstig og mindre gunstig organisatorisk kontekst. Logistisk regresjonsanalyse ble brukt for å undersøke trekk ved den organisatoriske konteksten på individ- og anleggsnivå.

**Resultater:** Førtifem prosent av utvalget (n= 453) opplevde å jobbe i en mer gunstig organisatorisk kontekst. Kontekstuelle trekk slik som en støttende arbeidskultur, flere evalueringsmekanismer og mer organisatoriske slakke ressurser, så ut til å forbedre individ- og fasilitetskonteksten, og indikerte dermed et mer gunstig arbeidsmiljø. Den logistiske regresjonsanalysen viste at helsepersonell i mer gunstige organisatoriske fasiliteter hadde nesten 2,7 ganger større sannsynlighet for å oppleve et bedre arbeidsmiljø totalt sett. Videre har individuelle trekk som å ha et annet morsmål enn norsk, jobbe dagskift, jobbe fulltid og yngre alder, betydelig økt sannsynligheten for å oppleve et gunstigere arbeidsmiljø, etter å ha tatt hensyn til fasilitetkonteksten.

**Konklusjon:** Organisatoriske kontekstuelle trekk, spesielt arbeidskulturen, evalueringsmekanismer og organisatorisk slakk ser ut til å forbedre det kontekstuelle arbeidsmiljøet på sykehjem. I tillegg kan det å tilby fulltidsarbeid og dag-/kveldsvakter, når det er mulig, være viktige individuelle arbeidsmiljøtrekk som har potensial til å forbedre arbeidsmiljøet, øke arbeidsgleden og sikre best-praksis omsorg.

## #6 Implementering av Tuning into Kids i norske barnehager. En undersøkelse av implementeringsprosesser i en grupperandomisert studie.

### Nygaard, E., Edvoll, M., Havighurst, S. S. Psykologisk Institutt, Universitetet i Oslo

**Bakgrunn:** Barnehager er en viktig arena for universelle helsefremmende tiltak, spesielt i Norge hvor 90% av førskolebarn går i barnehager. Tuning into Kids (TIK) er et emosjonssosialiserende tiltak for foreldre som er tilpasset barnehagepersonell. Det er mangel på kunnskap om implementeringsmekanismer. I denne studien undersøker vi sammenhenger mellom implementeringsfaktorer, implementeringsutfall og effekt av TIK i norske barnehager.

**Metode:** TIK ble implementert i norske FUS-barnehager, randomisert til intervensjons- (n=22) og kontrollbetingelse (n=27). Implementeringsstudien inkluderer informasjon fra 21 ledere og 265 barnehagelærere i intervensjonsgruppen. Implementeringsfaktorer basert på rammeverket Consolidated Framework for Implementation Research (CFIR) og implementeringsutfall basert på rammeverket Implementation Outcome Framework (IOF) ble målt med spørreskjemaer. Effekt av intervensjonen ble målt med spørreskjemaer og direkte observasjon.

**Resultater**: Deltagerne rapporterte mer positivt om individers holdninger til tiltaket enn for organisatoriske implementeringsfaktorer, for eksempel kommunikasjon mellom ledelse og ansatte. De ansattes holdninger til tiltaket var også høyest relatert til implementeringsutfall. Det var imidlertid ingen tydelig sammenheng mellom forhold ved implementeringen og effekt av intervensjonen.

**Konklusjoner:** I dette tiltaket var det svært positive holdninger til TIK blant de ansatte, mens det var mer skepsis til organisatoriske strukturer. Tilsvarende var de ansattes holdninger i større grad enn organisatoriske faktorer relatert til implementeringsutfall som fidelitet og rekkevidde. Metodiske begrensninger kan ha medvirket til mangel av tydelig sammenheng mellom implementering og den positive effekten av intervensjonen. På tross av at CFIR omhandler hva som påvirker implementeringen, mens IOF handler om konsekvensene av implementeringen, inkluderer begge rammeverkene personers vurderinger av intervensjonen. Fremtidige implementeringsstudier bør ivareta transaksjonelle perspektiver.

## #7 Bærekraftig implementering av høyintensiv gangtrening for pasienter til rehabilitering etter hjerneslag

### J.M. Halvorsen, C. Henderson, M.G. Hågå, K. Bergseth, M. Byhring, T.L.B. Eggen, H. Gustavsen, I. Rosseland, J.E. Nordvik, T. G. Hornby & J.L. Moore, Oslo kommune, Forsterket Rehabilitering, Aker

**Innledning:** I FIRST-Oslo prosjektet ble høyintensiv gangtrening (HIGT) implementert i klinisk praksis ved to rehabiliteringsavdelinger i Oslo gjennom bruk av rammeverket Knowledge-to-Action (KTA). Implementeringen resulterte i betydelig forbedring på relevante utfallsmål, og intervensjonen ble innført som standard behandling ved rehabiliteringsavdelingene. Etter vellykket implementering er videre monitorering en viktig del av prosessen, og utgjør den siste fasen i KTA modellen. Monitoreringen gjøres for å kunne vurdere om ny praksis opprettholdes over tid. Presentasjonen tar for seg strategier brukt for å sikre bærekraftig implementering av HIGT, samt resultatene av en bærekraftighetsanalyse utført to år etter opprinnelig implementering.

**Metodebeskrivelse:** Presentasjonen viser strategier benyttet for å fremme bærekraftig implementering, med utgangspunkt i de tre domenene i «NHS Sustainability Model». Modellen brukes også til å evaluere strategiene og for å kartlegge forbedringsområder. Deretter tar presentasjonen for seg en analyse av bærekraftigheten til den opprinnelige implementeringen av HIGT. Bærekraftig praksis ble definert som opprettholdelse av gangrelatert trening og skrittaktivitet, høy kardiovaskulær intensitet, samt tilsvarende forbedring av funksjonelle utfallsmål.

**Resultater:** Tretten ulike strategier ble benyttet for å fremme bærekraftig implementering. Disse presenteres ut i fra respektive domener i «NHS Sustainability Model». Bærekraftighetsanalysen viste en lavere skrittaktivitet to år etter implementeringen, dog var forskjellen ikke statistisk signifikant. Kardiovaskulær intensitet i behandlingen og forbedring av funksjonelle utfallsmål var uendret.

**Diskusjon og konklusjon**: Resultatene av bærekraftighetsanalysen indikerte opprettholdt mengde gangrelatert trening, skrittaktivitet og kardiovaskulær intensitet i behandlingen to år etter implementeringen av HIGT. Behandlingen medførte også tilsvarende forbedring av funksjonelle utfallsmål, og således samme nytteverdi for pasientene ved avdelingen.

## #8 Utvikling og resultater av en vellykket implementeringsplan for høyintensiv gangtrening for pasienter etter hjerneslag

### Elisabeth Bø, Jennifer L. Moore, Anne Erichsen, Ingvild Rosseland, Joakim Halvorsen, Hanne Bratlie, T. George Hornby & Jan Egil Nordvik, Enhet for rehabilitering, OUS

**Innledning**: Høyintensiv gangtrening (HIGT) er en anbefalt intervensjon for personer med nedsatt gangfunksjon etter hjerneslag. Forskning indikerer dog at effektiv implementering av intervensjoner i klinisk praksis er en utfordring.

**Metode:** To rehabiliteringsenheter samarbeidet med et kunnskapstranslasjonssenter for å implementere HIGT i klinisk praksis. Data ble samlet inn under konvensjonell rehabilitering og etter implementering av HIGT. Vi utviklet en implementeringsplan ved hjelp av The Knowledge-to-Action cycle (KTA). Consolidated Framework for Implementation Research ble brukt til å identifisere barrierer og velge implementeringsstrategier. Ved å bruke et mixed-methods design med spørreundersøkelser, uformelle diskusjoner og dokumentasjon av praksis evaluerte vi tidligere og nåværende praksis, barrierer, resultater og opprettholdelse av HIGT over tid.

**Resultater:** En fler-komponents implementeringsplan med 26 implementeringsstrategier ble utviklet. Konvensjonelle tiltak for å forbedre gange inkluderte en kombinasjon av balanse, styrketrening og gangintervensjoner. Barrierer for bruk av HIGT inkluderte manglende kunnskap om intervensjonen, antakelser om ny praksis, evne til å tilpasse seg HIGT, ressurser og kultur. Spørreundersøkelser og uformelle diskusjoner identifiserte betydelige endringer i opplevd praksis og bruk av HIGT som følge av implementeringsplanen. Resultatene viste signifikant forbedring på gangrelatert utfallsmål og positive effekter på helsesystemet. Oppfølgingsundersøkelsen etter to år bekreftet at HIGT fortsatt ble gjennomført i praksis.

**Diskusjon og konklusjon:** Flere momenter var viktig for det gode resultatet; utvikling av en fler-komponents implementeringsplan, det var lettere enn forventet å fjerne eksisterende praksis, terapeutene og organisasjonen viste evne til og var klar for endringene som krevdes. Bruk av implementeringsrammeverk og en flere-komponent implementeringsstrategi kan øke effektiviteten i kliniske implementeringsprosjekter.

## #9 Undersøkelse av Ledelse og organisatorisk endring for implementering (LOCI) som en strategi for implementering av kunnskapsbasert praksis i psykisk helsetjeneste.

### Karina Egeland & Ane-Marthe Solheim Skar, Nasjonalt kunnskapssenter om vold og traumatisk stress

**Bakgrunn:** Til tross for at god ledelse ofte nevnes som en nødvendighet for å lykkes med implementeringen av kunnskapsbaserte behandlingsmetoder i helsetjenestene, er det lite forskning på hva god ledelse for implementering innebærer.

**Metodikk**: I denne studien ble det gjennomført en trinnvis klyngerandomisert kontrollert studie for å undersøke effektiviteten av en implementeringsstrategi kalt Ledelse og organisatorisk endring for implementering (LOCI). Totalt 47 psykiske helsetjenester for voksne og barn deltok. Klinikkledere og terapeuter fylte ut spørreskjemaer som vurderte ledelse og implementeringsklima ved oppstart og 4, 8, 12 og 16 måneder. Det ble antatt at LOCI ville være relatert til høyere terapeutvurdert implementeringsledelse, transformasjonsledelse og implementeringsklima når klinikken deltok i LOCI sammenlignet med perioden før LOCI-deltakelse.

**Resultater:** Resultatene bekreftet vår hypotese ved å demonstrere en signifikant økning i terapeutvurdert implementerings-, transformasjonsledelse og implementeringsklima etter klinikkenes oppstart i LOCI. Dette ble opprettholdt på alle målingstidspunktene. Før klinikkene deltok i LOCI var det en jevn nedgang i skårene.

**Diskusjon:** Dette er den første studien som tester LOCI-strategien i en ikke-amerikansk kontekst. Studien fremmer feltet ved å demonstrere effekten av LOCI-strategien på nøkkelfaktorer som er trukket fram som viktige for vellykket implementering av kunnskapsbasert praksis.

## #10 Systematisk oversikt over kunnskapstranslasjon i rehabiliteringsforskning.

### Julia Aneth Mbalilaki, Jenni Moore & Ian Graham, RKR, Sunnaas sykehus

**Innledning:** Knowledge-to-Action rammeverket (kunnskap til handlingsmodellen) er en systematisk kunnskapstranslasjons-metode for implementering av kunnskapsbasert praksis, med mål om å redusere gapet mellom forskning og praksis, ved hjelp av en trinnvis prosess med 7 faser.

**Hensikt:** Få oversikt over hvordan publiserte studier som har anvendt KTA rammeverket beskriver de ulike implementeringsfasene; identifisere aktiviteter utført i hver fase av KTA; og foreslå hvordan slike prosesser i rehabilitering kan forbedres.

**Metodebeskrivelse:** Litteratursøk i elektroniske databaser Google Scholar og PubMed, ble gjennomført frem til 31. desember 2019.

**Sammendrag av resultatene:** Totalt 49 artikler som rapporterte bruk av KTA i kliniske implementeringsprosjekter i rehabilitering ble inkludert i oppsummeringen.

**Diskusjon og konklusjon:** Oppsummeringen viser at implementering av kunnskapsbasert praksis ikke automatisk skjer som følge av ny evidens, men at det kreves målbevisst planlegging med stor innsats for å implementere og endre. Flere retningslinjer for kliniske spørsmål bør produseres, gjøres tilgjengelige og implementering bør følges av tett veiledning. Å inkludere all «stakeholders» fra klinikk og forskning når retningslinjer for klinisk praksis skal publiseres og lanseres. Viktig å se også hvordan intervensjon og evidens kan tilpasses slik at de er gjennomførbare i den lokale konteksten uten at man mister innhold og anbefaling. Potensielle barrierer for implementering på alle nivå, må avdekkes og løses på en systematisk måte. Godt samarbeid i forskjellige grupper av stakeholdere er også nevnt som en viktig nøkkel for å lykkes med en implementering. Bruk av standardiserte måleverketøy er også løftet frem som avgjørende faktorer for å løfte kvaliteten på implementering og vurdering.

## #11 Gjennomføring av en RCT i spesialisthelsetjenesten: Utfordringer og muligheter

### Trude Fredriksen, BUP Lillehammer/Otta SIHF/UIO

**Bakgrunn:** Det er behov for å etablere et evidensbasert tiltak for søsken som pårørende ved ulike hjelpeinstanser i Norge. Per i dag er det ingen systematiske og evidensbaserte tilbud til denne gruppen. SIBS-RCT er en randomisert kontrollert studie som har som primært mål å måle effekten av det kunnskapsbaserte gruppetiltaket SIBS på søskens psykiske helse. Hensikt med denne presentasjonen er å beskrive aktuelle utfordringer og muligheter erfart etter iverksetting av en RCT i spesialisthelsetjenesten.

**Metode:** Deltakere rekrutteres gjennom BUP og Habiliteringstjenesten for barn via behandlere de har kontakt med og informasjonshefter. Intervensjonen drives i ordinær arbeidstid og går over to halve arbeidsdager. Gruppeledere er behandlere ved poliklinikken som har fått opplæring i SIBS.

**Resultat/Erfaringer:** Datainnsamlingen startet i mai 2019 og 50 grupper er gjennomført med totalt 170 søsken. Utfordringer er tilgang på nødvendige ressurser som personell, tid, romfasiliteter, datautstyr og kontormateriell. Behandlere glemmer å tilby tiltaket. Vanskelig å rekruttere deltakere. Ofte er det behov for ekstra kontakt og informasjon før deltakere samtykker. Deltakere har manglende forståelse av tiltaket til tross for å ha mottatt mye informasjon. Gode tilbakemeldinger fra deltakere som har gjennomført gruppetiltaket.

**Konklusjon:** Nødvendige forutsetninger for å gjennomføre en randomisert kontrollert studie av et gruppetiltak i spesialisthelsetjenesten, er lederforankring på flere nivå, stedlig ansvarlig person på enheten, hyppig informasjon om og påminning av tiltaket i kollegagruppen.

## #12 Implementering av en integrert kunnskapsoversettelsesintervensjon i sykehjem: erfaringer fra praksisutviklingssykepleiere (practice development nurses)

### Trine Lise Steinskog, O. Tranvåg, M. Nortvedt, D. Ciliska & B. Graverholt, Høgskulen på Vestlandet

**Bakgrunn:** Praksisutviklingssykepleiere (practice development nurses; PDN) i norske sykehjem har et spesifikt ansvar for å overføre forskning til en stadig mer kompleks praksis. PDN-er er involvert som sluttbrukere i IMPAKT-intervensjonen (IMPlementation of Action to Knowledge Translation). Ved å bruke en integrert kunnskapsoversettelsestilnærming, deltok de i et utdanningsprogram skreddersydd for deres identifiserte behov. I en andre komponent brukte de læringen sin i tilrettelegging ved implementering av verktøyet National Early Warning Score (NEWS2). Målet med denne studien var å utforske PDNs erfaringer med å delta i en IKT-pedagogisk intervensjon, og hvordan de brukte læringen i planlegging, skreddersying og innledende implementering av NEWS2.

**Metode**: En kvalitativ eksplorativ studie basert på en fenomenologisk hermeneutisk metode. Vi gjennomførte ni dybdeintervjuer av PDN-ene og åtte ikke-deltakende observasjonssesjoner av implementeringsstrategien/leveransen.

**Resultater**: PDN-ene ga uttrykk for at utdanningsprogrammet dekket deres behov og økte deres forståelse av ledende kunnskapsoversettelse (KT). De rapporterte en overgang fra å operere i en "stor svart boks for implementering" til et profesjonelt og strukturert modus for KT. PDN-ene rapporterte økt kompetanse i KT og i deres evne til å involvere og samarbeide med andre i deres anlegg. Organisatoriske kontekstuelle faktorer utfordret deres KT-innsats og implementering av NEWS2.

**Konklusjon:** Denne studien viser at en IKT-tilnærming har potensialet til å fremme og forbedre personalkompetanser og NH-beredskap for KT. Individuell motivasjon og kompetanse ble imidlertid utfordret innenfor en organisasjonskultur som var mindre mottakelig for denne nye lederrollen og nivået av KT-aktivitet.

## #13 Valideringer av Implementation Leadership Scale, Implementation Climate Scale, Implementation Citizenship Behavior Scale og AIM-, IAM- og FIM-skalaene i en norsk setting for psykisk helsevern

### Nora Braathu, Randi Hovden Borge, Mathilde Endsjø & Nadina Peters, Nasjonalt kunnskapssenter om vold og traumatisk stress (NKVTS)

**Bakgrunn:** Implementering av evidensbasert praksis (EBP) er av avgjørende betydning i helseinstitusjoner. Vellykket implementering kan påvirkes av implementeringsledelse, implementeringsklima, implementeringsborgerskapsatferd (ansattes ekstrarolleatferd for å støtte EBP-implementeringen), og oppfatninger av EBP. Den nåværende studien kombinerer fire psykometriske studier på instrumenter som brukes til å måle disse konstruksjonene i sammenheng med EBP-implementering. Dette var første gang disse instrumentene ble validert i norsk sammenheng.

**Mål:** Målet med de nåværende studiene var å vurdere de psykometriske egenskapene til seks instrumenter som ble brukt ved implementering av EBP i et norsk helsevesen.

**Metode:** Følgende skalaer ble brukt: The Implementation Leadership Scale (ILS), The Implementation Climate Scale (ICS), The Implementation Citizenship Behavior Scale (ICBS) og Feasibility of Intervention Measure (FIM), Intervention Appropriateness Measure (IAM) og Acceptability of Intervention Measure (AIM). Alle instrumenter ble utsatt for bekreftende faktoranalyse.

**Resultater**: Alle kartleggingsskjemaene viste aksepterte psykometriske egenskaper.

**Konklusjoner**: Resultatene tyder på at ILS, ICS, ICBS og FIM, IAM, AIM er valide og pålitelige verktøy for å måle disse implementeringskonseptene i det norske helsevesenet.

## #14 Kort innføring i KTA Knowledge to Action rammeverket

### Stein Arne Rimehaug, RKR, Sunnaas sykehus

Knowledge-to-Action (KTA) rammeverket er ment å favne hele evidensgrunnlaget fra implementeringsvitenskap, og være en hjelp i å sikre en mer helhetlig kunnskapstranslasjonsprosess. Flere andre presentasjoner som er submittert for NIMP 2021 konferansen omhandler avsluttede prosjekter som har brukt dette rammeverket, så det vil være nyttig å også få med en kort presentasjon av selve rammeverket, visualisert i KTA modellen fraStraus, Tetroe & Graham sin bok fra 2013 «Knowledge Translation in Health Care: Moving from Evidence to Practice». Et lite antall andre implementeringsprosjekter i norsk helsetjeneste har blitt inspirert av en animert Youtube instruksjonsvideo om KTA rammeverket «Fra kunnskap til handling» fra RKR, med til nå 2600 visninger, som også er lagt ut å Kunnskapssenterets nettressurs om kunnskapstranslasjon. Denne animasjonsvideoen ble laget i 2014 av Stein Arne Rimehaug, som også vil presentere KTA rammeverket i en 10-15 minutter lang presentasjon, sammen med Julia Mbalilaki, også fra RKR, Regional Kompetansetjeneste for Rehabilitering. Sekundært kan dette presenteres som en poster, men det vil bli et langt bedre læringsutbytte og helhet av å kunne presentere dette i plenum i forbindelse med andre presentasjoner som omhandler KTA.

## #15 Søsken som pårørende: En kartlegging av forebyggende psykisk helsearbeid i norske kommuner og prospektiv aksept av det gruppebaserte tiltaket SIBS

### Yngvild B. Haukeland, Torun M. Vatne, Ann-Helen Kongshavn & Ragnhild Bang Nes, Psykologisk institutt, Universitetet i Oslo

**Bakgrunn:** Helsepersonell er pliktet til å ivareta søsken som pårørende, men vi vet lite om etterlevelse av dette i norske kommuner. SIBS er en kunnskapsbasert søskenintervensjon med formål å forebygge psykisk uhelse. For vellykket spredning av SIBS trengs kunnskap om kommuneansattes vurderinger av tiltaket og antatte implementeringshindre.

**Metode:** Spørreskjema ble høsten 2019 sendt til helsesykepleiere, kommunepsykologer og kommuneoverleger for å kartlegge identifisering av søsken, samarbeid med spesialisthelsetjenesten og eksisterende tilbud til søsken. Vi kartla videre prospektiv aksept av SIBS og analyserte åpne utsagn om tiltakets styrker og svakheter med en deduktiv innholdsanalyse ut fra Consolidated Framework for Implementation Research.

**Resultater:** 332 informanter (58,9% helsesykepleiere) fra 253 kommuner deltok. Identifisering av søsken ble rapportert å hovedsakelig skje tilfeldig (62,1%) og sjeldent systematisk (11,5%). Majoriteten (66%) rapporterte ingen eller lite samarbeid med spesialisthelsetjenesten om pårørende søsken. Tilbud ble oftest gitt av skolehelsetjenesten (83,7%). Individuell samtale etter forespørsel fra familien var vanligst (88,9%) og gruppesamtaler sjeldent (11,1%). SIBS’ kvalitet ble vurdert som god og gjennomførbarhet som moderat. Vi fant at oppfattede styrker hovedsakelig omhandlet tiltakets kjennetegn (f.eks. gjennomtenkt og systematisk), samt individkarakteristikker (f.eks. opplevd samsvar med egne verdier og erfaringsgrunnlag). Oppfattede svakheter omhandlet hovedsakelig kontekstuelle faktorer; særlig begrenset tilgang på deltakere og fagpersoner i små kommuner og manglende samarbeid på tvers av kommuner og tjenestenivå.

**Konklusjon**: Videre arbeid med pårørende søsken bør fokusere på samarbeid mellom kommune og spesialisthelsetjenesten for å sikre at søsken identifiseres. Interkommunalt samarbeid bør også tilstrebes for å sikre tilstrekkelige ressurser til at kunnskapsbaserte intervensjoner som SIBS kan implementeres.

## #16 En veileder for kunnskapstranslasjon og implementering – på norsk!

### Birgitte Graverholt, Hilde Strømme & Donna Ciliska, Høgskulen på Vestlandet

**Bakgrunn:** Kunnskapsbasert praksis har et godt fotfeste i Norge og internasjonalt og diskusjoner handler ikke lenger om man skal jobbe kunnskapsbasert, men hvordan få det til. Opplæring i kunnskapsbasert praksis har pågått i mange år, men denne har i mindre grad hatt fokus på de trinnene som handler om å implementere og evaluere praksis. Registered Nurses of Ontario (RNAO) har utviklet en omfattende og kunnskapsbasert veileder for implementering av anbefalinger fra forskning. Veilederen kan brukes for å informere implementering, og som grunnlag for undervisning og opplæring i implementering.

**Hensikt:** Å oversette en veileder for implementering av kunnskapsbaserte anbefalinger, til norsk.

**Metode:** Oversettelsen av «Toolkit: Implementation of Best Practice Guidelines” skjedde i tre trinn: Et profesjonelt oversettelsesbyrå gjorde den første oversettelsen. En person med ekspertise i kunnskapsbasert praksis gjennomgikk oversettelsen og sikret tilpassing til norsk og faglig kontekst. Til sist leste en sykepleier hele veilederen for å sikre relevans for klinisk arbeid.

**Resultat:** Veilederen «Verktøykasse: Implementering av kunnskapsbaserte retningslinjer» finnes nå på norsk og er tilgjengelig for alle i Norge, gjennom lenke fra www.kunnskapsbasertpraksis.no (Helsebiblioteket). Veilederen er bygget opp rundt kunnskap-til-handling rammeverket. Denne modellen har blitt brukt i et stort implementeringsprosjekt (IMPAKT) og også systematisk i utvikling av et 15 studiepoengs emne om implementering på Master i kunnskapsbasert praksis i helsefag, HVL.

**Konklusjon:** Kunnskapsbasert praksis er bare staffasje om det ikke integreres i klinisk praksis. Helsepersonell har lenge manglet gode ressurser som kan støtte implementering av ny kunnskap, mens undervisere i kunnskapsbasert praksis har undervurdert kompleksiteten av å implementere. Denne omfattende veilederen kan brukes i begge sammenhenger, enten i sin helhet, eller kapittelvis og bidra til at pasienter møter en kunnskapsbasert helsetjeneste.

## #17 IPIC-studien: Effekten av et tverrprofesjonelt læringsprogram for brukermedvirkning blant eldre personer i korttidsrehabilitering: En kvasi-eksperimentell studie

### Linda Aimée Hartford Kvæl, Oslo Metropolitan University

**Bakgrunn:** Kommunal korttidsrehabilitering (KKR) fungerer som en bro mellom sykehus og hjem for eldre personer med kompleks problematikk. Brukermedvirkning betyr at de som mottar hjelp har rett til å medvirke. På tross av at brukermedvirkning er en demokratisk rettighet og et politisk mål, viser forskning at eldre pasienter og deres pårørende i KKR ikke opplever tilstrekkelig involvering. Helsepersonell rapporterer likeledes at brukermedvirkning er vanskelig å få til i den kliniske hverdagen. Det er således behov for økt kunnskap om hvordan implementere brukermedvirkning i KKR.

**Metode:** Gjennom IPIC-studien er målet å utvikle og evaluere en læringsintervensjon for helsepersonell i KKR basert på tverrfaglig simuleringsmetodikk. Undervisningsopplegget vil ta utgangspunkt i evidensbaserte kritiske punkter for brukermedvirkning i denne konteksten: 1) innkomsten 2) den tverrfaglige oppstartssamtalen og kontekstualisering av "Hva er viktig for deg?" 3) samarbeid bydel-helsehus 4) gode måltidsopplevelser 5) et rehabiliterende miljø og 6) utskrivelsen til hjemmet. Som del av undervisningsopplegget vil vi utvikle en kortfilm basert på real-life scenarioer som setter søkelyset på hvordan brukermedvirkning faktisk kan fremmes i den kliniske hverdagen. Læringsintervensjonen skal utvikles i samarbeid med praksisfeltet og gjennomføres i én korttidsavdeling gjennom tre halvdagsseminarer og fire oppfølgingssamtaler og sammenlignes med en annen tilsvarende avdeling som kontroll.

**Resultater:** I tillegg til å prosessevaluere læringsintervensjonen bant deltakerne vil vi vurdere effekten på brukermedvirkning, fysisk funksjon, pasienttilfredshet og pårørendebelastning. Økt kunnskap og bevissthet hos helsepersonell er en viktig implementeringsstrategi. Brukermedvirkning er assosiert med bedre utfall av rehabiliteringen, økt pasienttilfredshet, styrket autonomi og livskvalitet. Målet er økt implementering av brukermedvirkning i KKR.

## #18 Oversettelse og validering av Alberta Context Tool for bruk i norske sykehjem

### Jannicke Igland, Thomas Potrebny, Bente E. Bendixen, Anne Haugstvedt, Birgitte Espehaug, Kristine B. Titlestad & Birgitte Graverholt, Høgskulen på Vestlandet

**Bakgrunn:** Organisatorisk kontekst er anerkjent som viktig for å tilrettelegge for evidensbasert praksis og forbedre pasientresultater. Organisatorisk kontekst er et komplekst konstrukt å måle, og passende instrumenter som kan kvantifisere og måle kontekst er nødvendig.

**Hensikt:** Målet med denne studien var å oversette og tverrkulturelt tilpasse Alberta Context Tool (ACT) til norsk, og å teste reliabiliteten og strukturell validitet blant registrerte sykepleiere (RNer) og lisensierte praksissykepleiere (LPNs) som arbeider i sykehjem.

**Metoder:** Denne studien var en valideringsstudie som benyttet et tverrsnittsdesign. Utvalget besto av n = 956 helsepersonell fra 28 sykehjem fra en kommune i Norge. I den første fasen ble ACT oversatt før den ble administrert på 28 sykehjem. I den andre fasen ble intern konsistens og strukturell validitet utforsket ved hjelp av Cronbach’s alfa- og bekreftende faktoranalyse.

**Resultater:** En streng frem-og-bakoversettelsesprosess ble utført, utført av et team av akademikere, eksperter, profesjonelle oversettere og rettighetshaverne, før en akseptabel versjon av ACT ble testet og ferdigstilt. Den norske versjonen av ACT viste god intern konsistens med Cronbach’s alfa over .75 for alle konsepter bortsett fra Formal interactions hvor alfa var .69. Strukturell validitet var akseptabel for både RN-er og LPN-er med faktorladninger over 0,4 for de fleste varer.

**Konklusjoner:** Den norske versjonen av ACT er et gyldig mål på organisatorisk kontekst i norske sykehjem blant RNer og LPNer. Den norske versjonen av ACT kan derfor tjene som et viktig verktøy i fremtidige implementeringsstrategier og forskningsprosjekter.
