# Supplementary material for: Proceedings from the Second Annual Conference of the Norwegian Network for Implementation Research
Source: Glob Implement Res Appl. 2022 Nov 23;2(4):332–9. doi: 10.1007/s43477-022-00069-w (PMC9684746; doi:10.1007/s43477-022-00069-w)
Supplement: Supplementary file 2 — Supplementary file2 (DOCX 71 kb) [file 43477_2022_69_MOESM2_ESM.docx]

## ABSTRACTS IN ENGLISH

The NIMP conference

November 19, 2021

Oslo, Norway

[ABSTRACTS 1](#_Toc113194404)

[#2 Barriers and facilitators to implementation of the EU School Fruit and Vegetables Scheme: cross country study using the Consolidated Framework for Implementation Research (CFIR) 5](#_Toc113194405)

[Meshkovska, B., Forberger, S., Scheller, D. A., Wendt, J., Castellari, E., Tiboldo, G., Luszczynska, A., Lien, N. Department of Nutrition, University of Oslo 5](#_Toc113194406)

[#3 Development and testing of a web application utilized for evaluation of fidelity in implementation and process evaluation (IPE) 6](#_Toc113194407)

[Halvorsen, J. Læringsmiljøsenteret, University of Stavanger 6](#_Toc113194408)

[#4 The IMPAKT intervention in nursing homes – development, implementation, and evaluation of a complex intervention to meet relevant needs 7](#_Toc113194409)

[Graverholt, B., Espehaug, B, Potrebny, T., Igland, J., Ciliska, D. University college at Vestlandet 7](#_Toc113194410)

[#5 Organizational context in Norwegian nursing homes: a cross-sectional study 8](#_Toc113194411)

[Thomas Potrebny, Donna Ciliska, Birgitte Espehaug, Jannicke Igland & Birgitte Graverholt, University college at Vestlandet. 8](#_Toc113194412)

[#6 Implementing Tuning into Kids in Norwegian kindergartens. A study of implementation processes in a group-randomized study 9](#_Toc113194413)

[Nygaard, E., Edvoll, M., Havighurst, S. S. Department of Psychology, University of Oslo 9](#_Toc113194414)

[#7 Sustainable implementation of high-intensity gait training for patients in rehabilitation after stroke 10](#_Toc113194415)

[J.M. Halvorsen, C. Henderson, M.G. Hågå, K. Bergseth, M. Byhring, T.L.B. Eggen, H. Gustavsen, I. Rosseland, J.E. Nordvik, T. G. Hornby & J.L. Moore, Oslo municipality, unity of Rehabilitation, Aker 10](#_Toc113194416)

[#8 Development and results of a successful implementation plan for high-intensity gait training for patients after stroke 11](#_Toc113194417)

[Elisabeth Bø, Jennifer L. Moore, Anne Erichsen, Ingvild Rosseland, Joakim Halvorsen, Hanne Bratlie, T. George Hornby & Jan Egil Nordvik, rehabilitation unit, Oslo university hospital 11](#_Toc113194418)

[#9 Study of Leadership and Organizational Change for Implementation (LOCI) as a strategy for implementing evidence-based practice in mental health care 12](#_Toc113194419)

[Karina Egeland & Ane-Marthe Solheim Skar, Nasjonalt kunnskapssenter om vold og traumatisk stress 12](#_Toc113194420)

[#10 Systematic overview of knowledge translation in rehabilitation research 13](#_Toc113194421)

[Julia Aneth Mbalilaki, Jenni Moore & Ian Graham, RKR, Sunnaas hospital 13](#_Toc113194422)

[#11 Implementation of an RCT in the specialist health care service: Challenges and opportunities 14](#_Toc113194423)

[Trude Fredriksen, child and youth mental health, Lillehammer /Otta SIHF/University of Oslo 14](#_Toc113194424)

[#12 Implementing an integrated knowledge translation intervention in nursing homes: experiences of practice development nurses 15](#_Toc113194425)

[Trine Lise Steinskog, O. Tranvåg, M. Nortvedt, D. Ciliska & B. Graverholt, University college at Vestlandet 15](#_Toc113194426)

[#13 Validations of the Implementation Leadership Scale, Implementation Climate Scale, Implementation Citizenship Behaviour Scale and the AIM, IAM and FIM scales in a Norwegian mental health care setting 16](#_Toc113194427)

[Nora Braathu, Randi Hovden Borge, Mathilde Endsjø & Nadina Peters, Norwegian Centre for Violence and Traumatic Stress Studies (NKVTS) 16](#_Toc113194428)

[#14 Brief introduction to the KTA Knowledge to Action framework 17](#_Toc113194429)

[Stein Arne Rimehaug, RKR, Sunnaas hospital 17](#_Toc113194430)

[#15 Siblings of children with chronic disorders: A survey of preventive mental health work in Norwegian municipalities and prospective acceptance of the group-based measure SIBS 18](#_Toc113194431)

[Yngvild B. Haukeland, Torun M. Vatne, Ann-Helen Kongshavn & Ragnhild Bang Nes, Department of Psychology, University of Oslo 18](#_Toc113194432)

[#16 A guide for knowledge translation and implementation – in Norwegian! 19](#_Toc113194433)

[Birgitte Graverholt, Hilde Strømme & Donna Ciliska, University college at Vestlandet 19](#_Toc113194434)

[#17 The IPIC study: The effect of an interprofessional learning program on user participation among older people in short-term rehabilitation: A quasi-experimental study 20](#_Toc113194435)

[Linda Aimée Hartford Kvæl, Oslo Metropolitan University 20](#_Toc113194436)

[#18 Translation and validation of the Alberta Context Tool for use in Norwegian nursing homes 21](#_Toc113194437)

[Jannicke Igland, Thomas Potrebny, Bente E. Bendixen, Anne Haugstvedt, Birgitte Espehaug, Kristine B. Titlestad & Birgitte Graverholt, University college at Vestlandet 21](#_Toc113194438)

[ABSTRACTS IN NORWEGIAN 22](#_Toc113194439)

[#2 Barrierer og fasilitatorer for implementeringen av EU sin skolefrukt- og grønnsaksordning: tverrnasjonal studie som benytter Consolidated Framework for Implementation Research (CFIR) 23](#_Toc113194440)

[Meshkovska, B., Forberger, S., Scheller, D. A., Wendt, J., Castellari, E., Tiboldo, G., Luszczynska, A., Lien, N. Department of Nutrition, Universitetet i Oslo 23](#_Toc113194441)

[#3 Refleksjoner omkring utvikling og testing av app til bruk for måling av fidelitet i implementering og prosessevaluering (IPE) 24](#_Toc113194442)

[Halvorsen, J. Læringsmiljøsenteret, Universitetet i Stavanger 24](#_Toc113194443)

[#4 IMPAKT intervensjonen i sykehjem – utvikling, implementering og evaluering av en kompleks intervensjon for å møte relevante behov 25](#_Toc113194444)

[Graverholt, B., Espehaug, B, Potrebny, T., Igland, J., Ciliska, D. Høgskulen på Vestlandet 25](#_Toc113194445)

[#5 Organisasjons kontekst I Norske sykehjem: Ett tverrsnitt studie. 26](#_Toc113194446)

[Thomas Potrebny, Donna Ciliska, Birgitte Espehaug, Jannicke Igland & Birgitte Graverholt, Høgskulen på Vestlandet. 26](#_Toc113194447)

[#6 Implementering av Tuning into Kids i norske barnehager. En undersøkelse av implementeringsprosesser i en grupperandomisert studie. 27](#_Toc113194448)

[Nygaard, E., Edvoll, M., Havighurst, S. S. Psykologisk Institutt, Universitetet i Oslo 27](#_Toc113194449)

[#7 Bærekraftig implementering av høyintensiv gangtrening for pasienter til rehabilitering etter hjerneslag 28](#_Toc113194450)

[J.M. Halvorsen, C. Henderson, M.G. Hågå, K. Bergseth, M. Byhring, T.L.B. Eggen, H. Gustavsen, I. Rosseland, J.E. Nordvik, T. G. Hornby & J.L. Moore, Oslo kommune, Forsterket Rehabilitering, Aker 28](#_Toc113194451)

[#8 Utvikling og resultater av en vellykket implementeringsplan for høyintensiv gangtrening for pasienter etter hjerneslag 29](#_Toc113194452)

[Elisabeth Bø, Jennifer L. Moore, Anne Erichsen, Ingvild Rosseland, Joakim Halvorsen, Hanne Bratlie, T. George Hornby & Jan Egil Nordvik, Enhet for rehabilitering, OUS 29](#_Toc113194453)

[#9 Undersøkelse av Ledelse og organisatorisk endring for implementering (LOCI) som en strategi for implementering av kunnskapsbasert praksis i psykisk helsetjeneste. 30](#_Toc113194454)

[Karina Egeland & Ane-Marthe Solheim Skar, Nasjonalt kunnskapssenter om vold og traumatisk stress 30](#_Toc113194455)

[#10 Systematisk oversikt over kunnskapstranslasjon i rehabiliteringsforskning. 31](#_Toc113194456)

[Julia Aneth Mbalilaki, Jenni Moore & Ian Graham, RKR, Sunnaas sykehus 31](#_Toc113194457)

[#11 Gjennomføring av en RCT i spesialisthelsetjenesten: Utfordringer og muligheter 32](#_Toc113194458)

[Trude Fredriksen, BUP Lillehammer/Otta SIHF/UIO 32](#_Toc113194459)

[#12 Implementering av en integrert kunnskapsoversettelsesintervensjon i sykehjem: erfaringer fra praksisutviklingssykepleiere (practice development nurses) 33](#_Toc113194460)

[Trine Lise Steinskog, O. Tranvåg, M. Nortvedt, D. Ciliska & B. Graverholt, Høgskulen på Vestlandet 33](#_Toc113194461)

[#13 Valideringer av Implementation Leadership Scale, Implementation Climate Scale, Implementation Citizenship Behavior Scale og AIM-, IAM- og FIM-skalaene i en norsk setting for psykisk helsevern 34](#_Toc113194462)

[Nora Braathu, Randi Hovden Borge, Mathilde Endsjø & Nadina Peters, Nasjonalt kunnskapssenter om vold og traumatisk stress (NKVTS) 34](#_Toc113194463)

[#14 Kort innføring i KTA Knowledge to Action rammeverket 35](#_Toc113194464)

[Stein Arne Rimehaug, RKR, Sunnaas sykehus 35](#_Toc113194465)

[#15 Søsken som pårørende: En kartlegging av forebyggende psykisk helsearbeid i norske kommuner og prospektiv aksept av det gruppebaserte tiltaket SIBS 36](#_Toc113194466)

[Yngvild B. Haukeland, Torun M. Vatne, Ann-Helen Kongshavn & Ragnhild Bang Nes, Psykologisk institutt, Universitetet i Oslo 36](#_Toc113194467)

[#16 En veileder for kunnskapstranslasjon og implementering – på norsk! 37](#_Toc113194468)

[Birgitte Graverholt, Hilde Strømme & Donna Ciliska, Høgskulen på Vestlandet 37](#_Toc113194469)

[#17 IPIC-studien: Effekten av et tverrprofesjonelt læringsprogram for brukermedvirkning blant eldre personer i korttidsrehabilitering: En kvasi-eksperimentell studie 38](#_Toc113194470)

[Linda Aimée Hartford Kvæl, Oslo Metropolitan University 38](#_Toc113194471)

[#18 Oversettelse og validering av Alberta Context Tool for bruk i norske sykehjem 39](#_Toc113194472)

[Jannicke Igland, Thomas Potrebny, Bente E. Bendixen, Anne Haugstvedt, Birgitte Espehaug, Kristine B. Titlestad & Birgitte Graverholt, Høgskulen på Vestlandet 39](#_Toc113194473)

## #2 Barriers and facilitators to implementation of the EU School Fruit and Vegetables Scheme: cross country study using the Consolidated Framework for Implementation Research (CFIR)

### Meshkovska, B., Forberger, S., Scheller, D. A., Wendt, J., Castellari, E., Tiboldo, G., Luszczynska, A., Lien, N. Department of Nutrition, University of Oslo

**Background:** Since 2009/2010, the EU School Fruit and Vegetables Scheme has been implemented across most of the EU. It originates in the Common Agricultural Policy and has three main actions: delivery of fruit and vegetables to children in schools, accompanying educational measures and information.

**Aim:** The aim of this study is to identify barriers and facilitators to implementation of the Scheme based on perceptions from those responsible at government level and consider the applicability of the Consolidated Framework for Implementation Research for this purpose.

**Method:** Twenty-three semi-structured interviews (n.29) were conducted with persons from ministries of agriculture, health, and education, across 10 EU member states and with a representative from the EU level. Qualitative data was initially coded inductively, and subsequently inductive codes were mapped to the domains/constructs/sub-constructs of the CFIR. The country level was considered the inner setting of the CFIR. Barriers and facilitators were subsequently identified within each construct/subcontract.

**Results:** The following CFIR constructs (and barriers and facilitators linked to each) were found relevant: 1) Scheme characteristics domain: ‘Scheme source’, ‘evidence strength and quality’, ‘relative advantage’, ‘adaptability’, ‘design quality and packaging’ and ‘cost’; 2) outer setting: ‘cosmopolitanism’, ‘peer pressure’ and ‘external policy and incentives’; 3) inner setting: ‘structural characteristics’, ‘networks and communications’, ‘implementation climate’ and ‘readiness for implementation’; 4) characteristics of individuals: ‘knowledge and beliefs about the intervention’; 5) process: ‘engagement’, ‘executing’ and ‘reflecting and evaluating’.

**Conclusion:** CFIR is appropriate for use at country level, for identifying barriers and facilitators to policy implementation.

## #3 Development and testing of a web application utilized for evaluation of fidelity in implementation and process evaluation (IPE)

### Halvorsen, J. Centre for Learning Environment, University of Stavanger

My Ph.D.- project is a part of the RCT study Resilient where the aim is to improve students´ well-being and motivation, reduce emotional distress and loneliness, and improve academic outcomes. My dissertation has the tentative title «Evaluating Fidelity and Dosage in the Resilient-intervention», where the intervention is a social and emotional learning (SEL) curriculum consisting of 25 lessons in the following five core components: social relationship skills, mindfulness, problem-solving, emotion regulation and growth mindset. A primary focus in the dissertation is evaluating fidelity (e.g., if the intervention is implemented as planned) using a «web-based fidelity application» (further referred to as the app), developed as a tool for this purpose. The app is utilized by the implementers (teachers) to report the completion of lessons when implementing the curriculum in their own classrooms.

In this presentation, the focus is on the following research question: «How do the teachers assess the usability of the web-based fidelity application? », where the aim is to evaluate the usability of the app, to understand if, and possibly how, the app may be utilized as such a tool also in other research projects. This is particularly interesting in relation to projects where it is necessary to ensure high fidelity and implementation quality in implementation and process evaluation. The Ph.D.-project has a mixed methods design and is qualitatively driven, where data collection is conducted using focus group interviews, individual interviews, observations, and data from the app. Data from a pilot project is presented here. The data is collected from a digital focus group interview conducted on Zoom, with n=7 teachers who piloted the app in their own classrooms prior to the initiation of the intervention. The teachers, who are not taking part in the intervention, are recruited from across Norway. The app is inspired by a fidelity checklist as well as the theoretical framework on which the Resilient-intervention builds upon. The teachers tested a sample of lessons from the intervention (5 lessons: 1 lesson from each of the five core components). The teachers then registered to what extent the lessons were completed as planned, acquiring experience testing the app before taking part in the focus group interview. The primary aim of the interview was to collect information on the teachers' experiences and insight related to the app´s usability.

Preliminary analysis shows that the app can be a useful tool both for teachers and researchers in such interventions. Preliminary results also point to the potential for improvement in the app, such as a desire for clearer instructions before use and a need for a comment section for feedback while utilizing it. The knowledge and insight provided by the teachers have contributed to important perspectives from the field of practice related to how an app based on a fidelity checklist, can serve as a useful tool in quality assessment and quality development in IPE. The Ph.D.- project thus has the potential to contribute to closing the knowledge gaps related to evaluating fidelity in IPE, across several relevant research areas and research fields.

## #4 The IMPAKT intervention in nursing homes – development, implementation, and evaluation of a complex intervention to meet relevant needs

### Graverholt, B., Espehaug, B, Potrebny, T., Igland, J., Ciliska, D. Western Norway University of Applied Science

**Background:** The nursing home health care setting and workplace is becoming increasingly complex in line with new tasks and stricter requirements. This demands that professions in nursing homes must adjust their practice and competencies at high pace. But little is known about the challenges this sector faces to ensure that the development is evidence-based.

**Purpose:** To develop and implement an intervention that answers a relevant clinical question and competence learning needs in evidence-based practice in nursing homes.

**Method:** Methodological frameworks that form the basis of the study are the MRC framework of complex interventions and Integrated Knowledge Translation. To develop the intervention, we assessed clinical knowledge gaps and competence needs in nursing homes, using qualitative and quantitative methods. We explored the roles and responsibilities for evidence-based practice through interviews with managers at different levels. A needs assessment relevant to EBP competencies formed the basis for the intervention.

**Result:** We developed a cluster randomized controlled study among 19 nursing homes, informed by needs expressed by the sector itself. Through various sub-studies, we informed important decisions for the intervention. We decided upon one clinically important area in need of improvement, who should participate in the intervention, contextual considerations, and active ingredients of the intervention. Conclusion: An evidence-based development of NH practice requires that the entire organization is harmonized, with clarified roles and the right competence. The close collaboration between researchers and a nursing home organization in IMPAKT has led to several mutual benefits. These include a relevant intervention informed by those who work there, and easier and better recruitment of participants for studies.

## #5 Organizational context in Norwegian nursing homes: a cross-sectional study

### Thomas Potrebny, Donna Ciliska, Birgitte Espehaug, Jannicke Igland & Birgitte Graverholt, Western Norway University of Applied Science

**Background:** Favorable healthcare organizational context (i.e.: the work environment) is associated with better patient outcomes and increased job satisfaction. Long-term care is often considered a challenging work environment characterized by high job demands, low job control, high work pace and regular exposure to threats and violence which may affect patient care and increase staff turnover. This study aims to explore the features of a favorable organizational context in Norwegian nursing homes.

**Methods:** This study is a cross-sectional study of registered nurses and licensed practical nurses in Bergen, Norway (n = 1014). Organizational context was measured by the Alberta Context Tool. K-means clustering algorithm was used to differentiate between favorable and less favorable organizational context. Logistic regression analysis was used to examine features of organizational context at the individual and facility level.

**Results:** Forty five percent of the sample (n= 453) experienced working in a more favorable organizational context. Contextual features such as a supportive work culture, more evaluation mechanisms, and more organizational slack resources, appeared to improve individual and facility context, thus indicative of a more favorable work environment. The logistic regression analysis showed that healthcare workers in more favorable organizational context facilities, were almost 2.7 times more likely to experience a better work environment overall. Furthermore, individual features such as having another mother tongue than Norwegian, working day shifts, working full time and younger age, significantly increased the likelihood experiencing a more favorable work environment, after accounting for facility context.

**Conclusion:** Organizational contextual features, in particular the work culture, evaluation mechanisms and organizational slack appear to improve contextual work environment in nursing homes. In addition, offering full time employment and day/evening shifts, when possible, may be important individual work environment features that have the potential of improving the work environment, increase job satisfaction and ensure best-practice care.

## #6 Implementing Tuning into Kids in Norwegian kindergartens. A study of implementation processes in a group-randomized study

### Nygaard, E., Edvoll, M., Havighurst, S. S. Department of Psychology, University of Oslo

**Background:** Kindergartens are an important arena for universal health promotion measures, especially in Norway where 90% of pre-school children attend kindergartens. Tuning in to Kids (TIK) is an emotion socialization program for parents that is adapted to kindergarten staff. There is a lack of knowledge about implementation mechanisms. In this study, we examine connections between implementation factors, implementation outcomes and effect of TIK in Norwegian kindergartens.

**Method:** TIK was implemented in Norwegian FUS kindergartens, randomized to intervention (n=22) and control condition (n=27). The implementation study includes information from 21 leaders and 265 kindergarten teachers in the intervention group. Implementation factors based on the framework Consolidated Framework for Implementation Research (CFIR) and implementation outcomes based on the framework Implementation Outcome Framework (IOF) were measured with questionnaires. The effect of the intervention was measured with questionnaires and direct observation.

**Results:** Participants reported more positively about individuals' attitudes towards the program than about organizational implementation factors, for example communication between management and employees. The employees' attitudes towards the initiative were also most closely related to implementation outcomes. However, there was no clear connection between conditions during the implementation and the effect of the intervention.

**Conclusions:** In this measure, there were very positive attitudes towards TIK among the employees, while there was more skepticism towards organizational structures. Correspondingly, the employees' attitudes were to a greater extent than organizational factors related to implementation outcomes such as fidelity and penetration. Methodological limitations may have contributed to the lack of a clear connection between implementation and the positive effect of the intervention. Even though CFIR deals with what affects implementation, while IOF deals with the consequences of implementation, both frameworks include people's assessments of the intervention. Future implementation studies should consider transactional perspectives.

## #7 Sustainable implementation of high-intensity gait training for patients in rehabilitation after stroke

### J.M. Halvorsen, C. Henderson, M.G. Hågå, K. Bergseth, M. Byhring, T.L.B. Eggen, H. Gustavsen, I. Rosseland, J.E. Nordvik, T. G. Hornby & J.L. Moore, Oslo municipality, unity of Rehabilitation, Aker

**Introduction:** In the FIRST-Oslo project, high-intensity walking training (HIGT) was implemented in clinical practice at two rehabilitation departments in Oslo using the Knowledge-to-Action (KTA) framework. The implementation resulted in significant improvement on relevant outcome measures, and the intervention was introduced as standard treatment in the rehabilitation departments. After a successful implementation, further monitoring is an important part of the process, and constitutes the last phase in the KTA model. The monitoring is done to be able to assess whether new practices are maintained over time. The presentation deals with strategies used to ensure sustainable implementation of HIGT, as well as the results of a sustainability analysis carried out two years after initial implementation.

**Method description:** The presentation shows strategies used to promote sustainable implementation, based on the three domains in the "NHS Sustainability Model". The model is also used to evaluate the strategies and to map areas for improvement. The presentation then deals with an analysis of the sustainability of the original implementation of HIGT. Sustainable practice was defined as maintaining gait-related exercise and step activity, high cardiovascular intensity, as well as a corresponding improvement in functional outcome measures.

**Results:** Thirteen different strategies were used to promote sustainable implementation. These are presented based on respective domains in the "NHS Sustainability Model". The sustainability analysis showed a lower step activity two years after the implementation, however the difference was not statistically significant. Cardiovascular intensity in the treatment and improvement in functional outcome measures were unchanged. Discussion and conclusion: The results of the sustainability analysis indicated maintained amount of gait-related exercise, step activity and cardiovascular intensity in the treatment two years after the implementation of HIGT. The treatment also resulted in a corresponding improvement in functional outcome measures, and thus the same utility value for the patients in the ward.

## #8 Development and results of a successful implementation plan for high-intensity gait training for patients after stroke

### Elisabeth Bø, Jennifer L. Moore, Anne Erichsen, Ingvild Rosseland, Joakim Halvorsen, Hanne Bratlie, T. George Hornby & Jan Egil Nordvik, rehabilitation unit, Oslo university hospital

**Introduction:** High intensity walking training (HIGT) is a recommended intervention for people with reduced walking function after a stroke. Research indicates, however, that effective implementation of interventions in clinical practice is a challenge.

**Method:** Two rehabilitation units collaborated with a knowledge translation center to implement HIGT in clinical practice. Data were collected during conventional rehabilitation and after implementation of HIGT. We developed an implementation plan using The Knowledge-to-Action cycle (KTA). The "Consolidated Framework for Implementation Research" was used to identify barriers and select implementation strategies. Using a mixed-method design with surveys, informal discussions, and documentation of practice, we evaluated past and current practice, barriers, outcomes and maintenance of HIGT over time.

**Results:** A multi-component implementation plan with 26 implementation strategies was developed. Conventional interventions to improve walking included a combination of balance, strength training, and walking interventions. Barriers to using HIGT included lack of knowledge about the intervention, assumptions about new practice, ability to adapt to HIGT, resources and culture. Surveys and informal discussions identified significant changes in perceived practice and use of HIGT as a result of the implementation plan. The results showed significant improvement on gait-related outcome measures and positive effects on the health system. The follow-up survey after two years confirmed that HIGT was still carried out in practice.

**Discussion and conclusion:** Several aspects were important for the good result; development of a multi-component implementation plan, it was easier than expected to remove existing practices, the therapists and the organization showed the ability and were ready for the changes required. Using implementation frameworks and a multi-component implementation strategy can increase the effectiveness of clinical implementation projects.

## #9 Study of Leadership and Organizational Change for Implementation (LOCI) as a strategy for implementing evidence-based practice in mental health care

### Karina Egeland & Ane-Marthe Solheim Skar, Nasjonalt kunnskapssenter om vold og traumatisk stress

**Background:** Good leadership is often mentioned as a necessity for the successful implementation of evidence-based treatment methods in the health services. However, there is little research on what good leadership for implementation entails.

**Method:** A stepwise cluster randomized controlled trial was conducted to examine the effectiveness of an implementation strategy called Leadership and Organizational Change for Implementation (LOCI). A total of 47 mental health services for adults and children participated. Clinic leaders and therapists completed questionnaires assessing leadership and implementation climate at baseline and 4, 8, 12 and 16 months.

**Hypothesis:** It was hypothesized that LOCI would be related to higher therapist-rated implementation leadership, transformational leadership, and implementation climate when the clinics participated in LOCI compared to the period before LOCI participation.

**Results** The results confirmed our hypotheses by demonstrating a significant increase in therapist-rated implementation, transformational leadership and implementation climate after the clinics started in LOCI. This was maintained at all measurement times. Before the clinics participated in LOCI, there was a steady decline in the scores.

**Discussion:** This is the first study to test the LOCI strategy in a non-US context. The study advances the field by demonstrating the effect of the LOCI strategy on key factors that have been highlighted as important for the successful implementation of evidence-based practice.

## #10 Systematic overview of knowledge translation in rehabilitation research

### Julia Aneth Mbalilaki, Jenni Moore & Ian Graham, RKR, Sunnaas hospital

**Introduction:** The Knowledge-to-Action framework is a systematic knowledge translation method for the implementation of evidence-based practice, with the aim of reducing the gap between research and practice, using a step-by-step process with 7 phases.

**Purpose:** Get an overview of how published studies that have used the KTA framework describe the various implementation phases; identify activities carried out in each phase of the KTA; and suggest how such processes in rehabilitation can be improved.

**Method description:** A literature search in electronic databases Google Scholar and PubMed was carried out until 31 December 2019.

**Summary of the results:** A total of 49 articles reporting the use of KTA in clinical implementation projects in rehabilitation were included in the summary.

**Discussion and conclusion:** The summary showed that implementation of evidence-based practice does not automatically happen as a result of new evidence, but that purposeful planning with great effort is required to implement and change. More guidelines for clinical questions should be produced, made available and implementation should be followed by close supervision. To include all "stakeholders" from the clinic and research when guidelines for clinical practice are to be published and launched. It is also important to see how intervention and evidence can be adapted so that they are feasible in the local context without losing content and recommendation. Potential barriers to implementation at all levels must be uncovered and solved in a systematic way. Good cooperation in different groups of stakeholders is also mentioned as an important key to a successful implementation. The use of standardized measurement tools has also been highlighted as a decisive factor in raising the quality of implementation and assessment.

## #11 Implementation of an RCT in the specialist health care service: Challenges and opportunities

### Trude Fredriksen, child and youth mental health, Lillehammer, Innlandet Hospital Health Trust, University of Oslo

**Background:** There is a need to establish evidence-based interventions for siblings as next of kin at health care agencies in Norway. The SIBS-RCT is a randomized controlled study where the primary aim is to measure the effect of the evidence-based group intervention SIBS on siblings' mental health. The purpose of this presentation is to describe current challenges and opportunities experienced during implementing an RCT in the specialist health care service.

**Method:** Participants were recruited through BUP (Psychiatric outpatient clinic for children and adolescents) and the Habilitation Service for children via therapists and information booklets. The intervention was carried out during ordinary working hours. Group leaders were therapists at the outpatient clinic who had received training in SIBS intervention.

**Results/Experiences:** Data collection started in May 2019 and 50 groups have been completed with a total of 170 siblings. Challenges were access to necessary resources such as personnel, time, room facilities, computer equipment and office supplies. Recruitment of participants were difficult. Therapists often forgot to offer the intervention. There was often a need for additional information to the families. Participants had little understanding of the intervention despite having received information in advance. Participants gave overall positive feedback after the intervention.

**Conclusion:** To implement a randomized controlled study in the specialist health care service, there is a need for close collaboration with leaders at the clinics, at least one person at the clinic must have the responsibility and there is a need for frequent information about and reminders of the intervention to all therapists at the clinic.

## #12 Implementing an integrated knowledge translation intervention in nursing homes: experiences of practice development nurses

### Trine Lise Steinskog, O. Tranvåg, M. Nortvedt, D. Ciliska & B. Graverholt, Western Norway University of Applied Science

**Background**: Practice Development Nurses (PDNs) in Norwegian nursing homes hold a specific responsibility for transferring research into an increasingly complex practice. PDNs are involved as end-users in the IMPAKT (IMPlementation of Action to Knowledge Translation) intervention. By using an integrated knowledge translation approach, they participated in an educational program tailored to their identified needs. In a second component, they applied their learning in facilitation-upon implementation of the tool National Early Warning Score (NEWS2). The aim of this study was to explore the PDNs’ experiences of participating in an IKT educational intervention, and how they applied the learning in planning, tailoring and initial implementation of the NEWS2.

**Method:** A qualitative exploratory study based on a phenomenological hermeneutical method. We conducted nine in-depth interviews of the PDNs and eight non-participatory observational sessions of the implementation strategy/delivery.

**Results:** The PDNs expressed that the educational program met their needs and enhanced their understanding about leading knowledge translation (KT). They reported a move from operating in a “big black box of implementation” to a professional and structured mode of KT. The PDNs reported enhanced competencies in KT and in their ability to involve and collaborate with others in their facility. Organizational contextual factors challenged their KT efforts and implementation of the NEWS2.

**Conclusion**: This study demonstrates that an IKT approach has the potential to advance and improve staff competencies and NH readiness for KT. However, individual motivation and competencies were challenged within an organizational culture which was less receptive to this new leadership role and level of KT activity.

## #13 Validations of the Implementation Leadership Scale, Implementation Climate Scale, Implementation Citizenship Behaviour Scale and the AIM, IAM and FIM scales in a Norwegian mental health care setting

### Nora Braathu, Randi Hovden Borge, Mathilde Endsjø & Nadina Peters, Norwegian Centre for Violence and Traumatic Stress Studies (NKVTS)

**Background:** Evidence-based practice (EBP) implementation is of crucial importance in health care institutions. Successful implementation can be influenced by implementation leadership, implementation climate, implementation citizenship behavior (employees’ extra-role behaviors to support the EBP implementation), and perceptions of the EBP. The current study combines four psychometric studies on instruments used to measure these constructs in the context of EBP implementation. This was the first time these instruments were validated in a Norwegian context.

**Method:** The following scales were used: The Implementation Leadership Scale (ILS), The Implementation Climate Scale (ICS), The Implementation Citizenship Behavior Scale (ICBS), and the Feasibility (FIM), Appropriateness (IAM) and Acceptability (AIM) scales. All instruments were subjected to confirmatory factor analysis.

**Results:** All instruments showed acceptable psychometric properties.

**Conclusions:** The results suggest that the ILS, ICS, ICBS, and FIM, IAM, AIM are valid and reliable tools to measure these implementation concepts in a Norwegian health care setting.

## #14 Brief introduction to the KTA Knowledge to Action framework

### Stein Arne Rimehaug, RKR, Sunnaas hospital

The Knowledge-to-Action (KTA) framework is intended to embrace the entire evidence base from implementation science, and to be a help in ensuring a more holistic knowledge translation process. Several other presentations that have been submitted for the NIMP 2021 conference deal with completed projects that have used this framework, so it would be useful to also include a short presentation of the framework itself, visualized in the KTA model from Straus, Tetroe & Graham's book from 2013 « Knowledge Translation in Health Care: Moving from Evidence to Practice». A small number of other implementation projects in the Norwegian health service have been inspired by an animated YouTube instructional video about the KTA framework "From knowledge to action" from RKR, with 2,600 views to date, which has also been posted to the Knowledge Centre's online resource on knowledge translation. This animation video was made in 2014 by Stein Arne Rimehaug, who will also present the KTA framework in a 10-15-minute-long presentation, together with Julia Mbalilaki, also from RKR, Regional Competence Service for Rehabilitation. Secondarily, this can be presented as a poster, but there will be a far better learning outcome and totality from being able to present this in plenary in connection with other presentations dealing with KTA.

## #15 Siblings of children with chronic disorders: A survey of preventive mental health work in Norwegian municipalities and prospective acceptance of the group-based measure SIBS

### Yngvild B. Haukeland, Torun M. Vatne, Ann-Helen Kongshavn & Ragnhild Bang Nes, Department of Psychology, University of Oslo

**Background:** Health personnel are obliged to look after siblings of children with chronic disorders, but we know little about compliance with this in Norwegian municipalities. SIBS is an evidence-based sibling intervention with the aim of preventing mental illness. For the successful spread of SIBS, knowledge is needed about municipal employees' assessments of the measure and assumed implementation obstacles.

**Method:** In the autumn of 2019, a questionnaire was sent to public health nurses, municipal psychologists, and municipal chief doctors to survey the identification of siblings, cooperation with the specialist health service and existing services for siblings. We further mapped prospective acceptance of SIBS and analyzed open statements about the initiative's strengths and weaknesses with a deductive content analysis based on the Consolidated Framework for Implementation Research.

**Results**: 332 informants (58.9% public health nurses) from 253 municipalities participated. Identification of siblings was reported to happen mainly randomly (62.1%) and rarely systematically (11.5%). The majority (66%) reported no or little cooperation with the specialist health service regarding siblings of children with chronic disorders. Offers were most often given by the school health service (83.7%). Individual conversations at the request of the family were most common (88.9%) and group conversations were rare (11.1%). SIBS' quality was assessed as good and feasibility as moderate. We found that perceived strengths mainly dealt with the initiative's characteristics (e.g., well-thought-out and systematic), as well as individual characteristics (e.g., perceived conformity with own values ​​and experience). Perceived weaknesses dealt mainly with contextual factors, particularly limited access to participants and professionals in small municipalities and a lack of cooperation across municipalities and service levels.

**Conclusion:** Further work with siblings of children with chronic disorders should focus on cooperation between the municipality and the specialist health service to ensure that the siblings are identified. Inter-municipal cooperation should also be sought to ensure sufficient resources so that evidence-based interventions such as SIBS can be implemented.

## #16 A guide for knowledge translation and implementation – in Norwegian!

### Birgitte Graverholt, Hilde Strømme & Donna Ciliska, Western Norway University of Applied Science

**Background**: Evidence-based practice has a solid foothold in Norway and internationally. Still, we need discussions about how to make it happen. Training in evidence-based practice has been ongoing for many years, but with less emphasis on the steps of implementing and evaluating practice. Registered Nurses of Ontario (RNAO) has developed a comprehensive and evidence-based guide for implementing recommendations from research. The guide can be used to inform implementation, and as a basis for teaching and training in implementation.

**Purpose:** To translate a guide for the implementation of evidence-based recommendations into Norwegian.

**Method:** The translation of "Toolkit: Implementation of Best Practice Guidelines" took place in three steps: A professional translation agency did the first translation. A person with expertise in evidence-based practice reviewed the translation and ensured adaptation to the Norwegian and professional context. Finally, a nurse read the entire guide to ensure relevance for clinical work.

**Result:** The guide "Toolbox: Implementation of evidence-based guidelines" is now available in Norwegian and is available to everyone in Norway, through a link from www.kunnskapsbasertpraksis.no (Health Library). The guide is built around the knowledge-to-action framework. This model has been used in a large implementation project (IMPAKT) and also systematically in the development of a 15 credit course on implementation on the Master's in evidence-based practice in health sciences, HVL.

**Conclusion:** Evidence-based practice is only a fade if not integrated into clinical practice. Healthcare personnel have long lacked good resources that can support the implementation of new knowledge, while teachers in evidence-based practice have underestimated the complexity of implementation. This comprehensive guide can be used in both contexts, either in its entirety, or chapter by chapter and contribute to patients encountering evidence-based health services.

## #17 The IPIC study: The effect of an interprofessional learning program on user participation among older people in short-term rehabilitation: A quasi-experimental study

### Linda Aimée Hartford Kvæl, Oslo Metropolitan University

**Background:** Communal short-term rehabilitation (KKR) functions as a bridge between hospital and home for elderly people with complex problems. User participation means that those who receive help have the right to contribute. Even though user participation is a democratic right and a political goal, research shows that elderly patients and their relatives in KKR do not experience sufficient involvement. Healthcare professionals also report that user participation is difficult to achieve in clinical everyday life. There is thus a need for increased knowledge about how to implement user participation in KKR.

**Method:** Through the IPIC study, the aim is to develop and evaluate a learning intervention for healthcare personnel in KKR based on interdisciplinary simulation methodology. The teaching plan will be based on evidence-based critical points for user participation in this context: 1) the income 2) the interdisciplinary start-up conversation and contextualization of "What is important to you?" 3) cooperation between district health center 4) good meal experiences 5) a rehabilitative environment and 6) discharge to home. As part of the teaching program, we will develop a short film based on real-life scenarios that will focus on how user participation can be promoted in clinical everyday life. The learning intervention is to be developed in collaboration with the field of practice and carried out in one short-term department through three half-day seminars and four follow-up interviews and compared with another similar department as a control.

**Results**: In addition to process evaluation of the learning intervention among the participants, we will assess the effect on user participation, physical function, patient satisfaction and burden on relatives. Increased knowledge and awareness among healthcare personnel is an important implementation strategy. User participation is associated with better outcomes of the rehabilitation, increased patient satisfaction, strengthened autonomy and quality of life. The goal is increased implementation of user participation in KKR.

## #18 Translation and validation of the Alberta Context Tool for use in Norwegian nursing homes

### Jannicke Igland, Thomas Potrebny, Bente E. Bendixen, Anne Haugstvedt, Birgitte Espehaug, Kristine B. Titlestad & Birgitte Graverholt, Western Norway University of Applied Science

**Background:** Organizational context is recognized as important for facilitating evidence-based practice and improving patient outcomes. Organizational context is a complex construct to measure and appropriate instruments that can quantify, and measure context are needed.

**Purpose:** The aim of this study was to translate and cross-culturally adapt the Alberta Context Tool (ACT) to Norwegian, and to test the reliability and structural validity among registered nurses (RNs) and licensed practice nurses (LPNs) working in nursing homes.

**Methods:** This study was a validation study utilizing a cross-sectional design. The sample consisted of n = 956 healthcare personnel from 28 nursing homes from a municipality in Norway. In the first stage, the ACT was translated before being administered in 28 nursing homes. In the second stage, internal consistency and structural validity were explored using Cronbach’s alpha and confirmatory factor analysis.

**Results:** A rigorous forward-and-back translation process was performed including a team of academics, experts, professional translators, and the copyright holders, before an acceptable version of the ACT was piloted and finalized. The Norwegian version of the ACT showed good internal consistency with Cronbach’s alpha above .75 for all concepts except for Formal interactions where the alpha was .69. Structural validity was acceptable for both RNs and LPNs with factors loadings more than .4 for most items.

**Conclusions:** The Norwegian version of the ACT is a valid measure of organizational context in Norwegian nursing homes among RNs and LPNs. The Norwegian version of the ACT may therefore serve as an important tool in future implementation strategies and research projects.

## ABSTRACTS IN NORWEGIAN

The NIMP conference

November 19, 2021

Oslo, Norway

## #2 Barrierer og fasilitatorer for implementeringen av EU sin skolefrukt- og grønnsaksordning: tverrnasjonal studie som benytter Consolidated Framework for Implementation Research (CFIR)

### Meshkovska, B., Forberger, S., Scheller, D. A., Wendt, J., Castellari, E., Tiboldo, G., Luszczynska, A., Lien, N. Department of Nutrition, Universitetet i Oslo

**Bakgrunn:** Siden 2009/2010 har EUs skolefrukt- og grønnsaksordning vært implementert i store deler av EU. Den har sin opprinnelse EUs felles landbrukspolitikken og har tre hovedhandlinger: levering av frukt og grønnsaker til barn i skolen, medfølgende pedagogiske tiltak og informasjon.

**Mål:** Målet med denne studien er å identifisere barrierer og fasilitatorer for implementering av ordningen basert på oppfatninger fra de ansvarlige på myndighetsnivå og vurdere anvendeligheten av the Consolidated Framework for Implementation Research for dette formålet.

**Metode**: Tjuetre semistrukturerte intervjuer (N=29) ble gjennomført med personer fra landbruks-, helse- og utdanningsdepartementer, på tvers av 10 EU-medlemsland og med en representant fra EU-nivået. Kvalitative data ble opprinnelig kodet induktivt, og deretter ble den induktive kodingen kartlagt til domenene/konstruksjonene/subkonstruksjonene til CFIR. Det nasjonale nivået ble ansett som den indre rammen for CFIR. Barrierer og fasilitatorer ble deretter identifisert innenfor hver konstruksjon/subkonstruksjoner.

**Resultater:** Følgende CFIR-konstruksjoner (og barrierer og fasilitatorer knyttet til hver) ble funnet relevante: 1) Ordningens karakteristikker: 'ordningens kilder', 'bevisstyrke og kvalitet', 'relativ fordel', 'adaptabilitet', 'designkvalitet og emballasje' og 'kostnad'; 2) ytre setting: 'kosmopolitisme', 'gruppepress' og 'ekstern politikk og insentiver'; 3) indre setting: 'strukturelle karakteristikker', 'nettverk og kommunikasjon', 'implementeringsklima' og 'hvor klar man er for implementering'; 4) karakteristikker ved individer: 'kunnskap og tro om intervensjonen'; 5) prosess: 'engasjement', 'utføre' og 'reflektere og evaluere'.

**Konklusjon:** CFIR er egnet for bruk på nasjonalt nivå, for å identifisere barrierer og fasilitatorer for policyimplementering.

## #3 Refleksjoner omkring utvikling og testing av app til bruk for måling av fidelitet i implementering og prosessevaluering (IPE)

### Halvorsen, J. Læringsmiljøsenteret, Universitetet i Stavanger

Mitt PhD-prosjekt er en del av RCT-studien Resilient, hvor målet er å bedre studentenes velvære og motivasjon, redusere emosjonell stress og ensomhet, og bedre akademisk utfall. Min avhandling har tentative tittelen: «Evaluere fidelitet og dose i Resilient-intervensjonen», hvor intervensjonen består av sosiale og emosjonelle læringsmål (SEL) fordelt på 25 timer i følgende fem hovedkomponenter: sosiale relasjonsferdigheter, mindfulness, problemløsning, emosjonsregulering og vekst i tankesett. Et hovedfokus i avhandlingen er evaluering av fidelitet (dvs. om intervensjonen er implementert som planlagt) ved å bruke en webbasert fidelitetsapplikasjon (referert til som appen), utviklet som et verktøy for dette målet. Appen er benyttet av implementørene (lærerne) for å rapportere gjennomføringen av timene når de implementerer målene i klasserommene.

I denne presentasjonen vil fokuset være på følgende forskningsspørsmål: Hvordan måler lærerne bruken av den webbaserte fidelitetsappen? Målet er å evaluere bruken av appen, å forstå hvis, og om mulig hvordan appen kan benyttes som et verktøy også i andre prosjekter. Dette er spesielt interessant knyttet til prosjekter hvor det er nødvendig å måle fidelitet og implementeringskvalitet i implementering og prosessevaluering. PhD-prosjektet benytter blandet metode og er kvalitativt drevet, hvor datainnsamlingen gjennomføres med fokusgruppeintervjuer, individuelle intervjuer, observasjon og datainnsamling via appen. Data fra et pilotprosjekt vil presenteres her. Data er samlet inn fra et digitalt fokusgruppeintervju (zoom) med 7 lærere som piloterte appen deres klasserom før implementeringen startet opp. Lærerne, som ikke tar del I intervensjonen, er rekruttert fra hele Norge. Appen er inspirert av fidelitysjekklisten og det teoretiske rammeverket som Resilient-intervensjonen bygger på. Lærerne testet ut en del av timene fra intervensjonen (5 timer: 1 time fra hver av komponentene). Lærerne registrerte deretter i hvilken grad timene ble gjennomført som planlagt, noe som krevde erfaring med å teste ut appen før de deltok i fokusgruppeintervjuet. Hovedmålet for intervjuet var å samle inn informasjon fra lærernes erfaringer med å bruke appen.

Preliminære analyser viser at appen kan være nyttig verktøy både for lærerne og forskere knyttet til slike intervensjoner. Preliminære resultater også viser potensiale for forbedringer i appen, slik som et ønske om tydeligere instruksjoner før bruk, samt behov for felt hvor man kan legge inn kommentarer. Kunnskapen og innsikten fra lærerne har bidratt til viktige perspektiver fra praksisfeltet om bruk av app som måler fidelitet. Appen kan være et nyttig verktøy for å måle kvalitet og forbedre kvalitet av IPE. PhD-prosjektet har potensiale for å bidra til å lukke kunnskapsgapet relatert til å evaluere fidelitet av IPE, på tvers av relevante forskningsområder og forskningsfelt.

## #4 IMPAKT intervensjonen i sykehjem – utvikling, implementering og evaluering av en kompleks intervensjon for å møte relevante behov

### Graverholt, B., Espehaug, B, Potrebny, T., Igland, J., Ciliska, D. Høgskulen på Vestlandet

**Bakgrunn:** Sykehjem som helsetjenestesetting og arbeidssted blir stadig mer kompleks i tråd med nye oppgaver og skjerpete krav. Dette krever at profesjoner i sykehjem må utvikle praksis og kompetanse i høyt tempo. Men vi vet lite om hvilke utfordringer denne sektoren har for å sikre at utviklingen skal være kunnskapsbasert. Hensikt Å utvikle og implementere en intervensjon som svarer på kliniske spørsmål og kompetansebehov i kunnskapsbasert praksis i sykehjem.

**Metode:** Metodiske rammeverk som ligger til grunn for studien er MRC framework of complex interventions og Integrated Knowledge Translation. For å utvikle intervensjonen kartla vi kliniske kunnskapshull og kompetansebehov i sykehjem, gjennom kvalitative og kvantitative metoder. Vi utforsket rollefordeling og ansvar for kunnskapsbasert praksis gjennom intervjuer med ledere på ulike nivå. Behov rettet mot det å jobbe kunnnskapsbasert ble kartlagt

**Resultat:** Vi utviklet en klyngerandomisert kontrollert studie blant 19 sykehjem, informert av behov ytret fra sektoren selv. Gjennom ulike delstudier informerte vi viktige beslutninger for intervensjonen: Ett klinisk viktig område med behov for forbedring, deltakere, kontekstuelle hensyn og aktive komponenter.

**Konklusjon:** En kunnskapsbasert utvikling av tjenestene krever at hele organisasjonen harmoniseres, med avklarte roller og rett kompetanse. Det tette samarbeidet mellom forskere fra HVL og sykehjem som helsetjenestesetting i IMPAKT har ført til flere gjensidige fordeler. Disse inkluderer relevant forskning informert av de som jobber der, og enklere og bedre rekruttering av deltakere til studier.

## #5 Organisasjons kontekst I Norske sykehjem: Ett tverrsnitt studie.

### Thomas Potrebny, Donna Ciliska, Birgitte Espehaug, Jannicke Igland & Birgitte Graverholt, Høgskulen på Vestlandet.

**Bakgrunn:** Gunstig helseorganisasjonskontekst (dvs.: arbeidsmiljøet) er assosiert med bedre pasientresultater og økt arbeidstilfredshet. Langtidspleie anses ofte som et utfordrende arbeidsmiljø preget av høye jobbkrav, lav jobbkontroll, høyt arbeidstempo og regelmessig eksponering for trusler og vold som kan påvirke pasientbehandlingen og øke personalutskiftingen. Denne studien sitt mål er å utforske trekk ved en gunstig organisatorisk kontekst i norske sykehjem.

**Metoder:** Denne studien er en tverrsnittsstudie av registrerte sykepleiere og praktiserende sykepleiere med lisens i Bergen, Norge (n = 1014). Organisasjonskontekst ble målt med Alberta Context Tool. K-betyr klyngealgoritme ble brukt for å skille mellom gunstig og mindre gunstig organisatorisk kontekst. Logistisk regresjonsanalyse ble brukt for å undersøke trekk ved den organisatoriske konteksten på individ- og anleggsnivå.

**Resultater:** Førtifem prosent av utvalget (n= 453) opplevde å jobbe i en mer gunstig organisatorisk kontekst. Kontekstuelle trekk slik som en støttende arbeidskultur, flere evalueringsmekanismer og mer organisatoriske slakke ressurser, så ut til å forbedre individ- og fasilitetskonteksten, og indikerte dermed et mer gunstig arbeidsmiljø. Den logistiske regresjonsanalysen viste at helsepersonell i mer gunstige organisatoriske fasiliteter hadde nesten 2,7 ganger større sannsynlighet for å oppleve et bedre arbeidsmiljø totalt sett. Videre har individuelle trekk som å ha et annet morsmål enn norsk, jobbe dagskift, jobbe fulltid og yngre alder, betydelig økt sannsynligheten for å oppleve et gunstigere arbeidsmiljø, etter å ha tatt hensyn til fasilitetkonteksten.

**Konklusjon:** Organisatoriske kontekstuelle trekk, spesielt arbeidskulturen, evalueringsmekanismer og organisatorisk slakk ser ut til å forbedre det kontekstuelle arbeidsmiljøet på sykehjem. I tillegg kan det å tilby fulltidsarbeid og dag-/kveldsvakter, når det er mulig, være viktige individuelle arbeidsmiljøtrekk som har potensial til å forbedre arbeidsmiljøet, øke arbeidsgleden og sikre best-praksis omsorg.

## #6 Implementering av Tuning into Kids i norske barnehager. En undersøkelse av implementeringsprosesser i en grupperandomisert studie.

### Nygaard, E., Edvoll, M., Havighurst, S. S. Psykologisk Institutt, Universitetet i Oslo

**Bakgrunn:** Barnehager er en viktig arena for universelle helsefremmende tiltak, spesielt i Norge hvor 90% av førskolebarn går i barnehager. Tuning into Kids (TIK) er et emosjonssosialiserende tiltak for foreldre som er tilpasset barnehagepersonell. Det er mangel på kunnskap om implementeringsmekanismer. I denne studien undersøker vi sammenhenger mellom implementeringsfaktorer, implementeringsutfall og effekt av TIK i norske barnehager.

**Metode:** TIK ble implementert i norske FUS-barnehager, randomisert til intervensjons- (n=22) og kontrollbetingelse (n=27). Implementeringsstudien inkluderer informasjon fra 21 ledere og 265 barnehagelærere i intervensjonsgruppen. Implementeringsfaktorer basert på rammeverket Consolidated Framework for Implementation Research (CFIR) og implementeringsutfall basert på rammeverket Implementation Outcome Framework (IOF) ble målt med spørreskjemaer. Effekt av intervensjonen ble målt med spørreskjemaer og direkte observasjon.

**Resultater**: Deltagerne rapporterte mer positivt om individers holdninger til tiltaket enn for organisatoriske implementeringsfaktorer, for eksempel kommunikasjon mellom ledelse og ansatte. De ansattes holdninger til tiltaket var også høyest relatert til implementeringsutfall. Det var imidlertid ingen tydelig sammenheng mellom forhold ved implementeringen og effekt av intervensjonen.

**Konklusjoner:** I dette tiltaket var det svært positive holdninger til TIK blant de ansatte, mens det var mer skepsis til organisatoriske strukturer. Tilsvarende var de ansattes holdninger i større grad enn organisatoriske faktorer relatert til implementeringsutfall som fidelitet og rekkevidde. Metodiske begrensninger kan ha medvirket til mangel av tydelig sammenheng mellom implementering og den positive effekten av intervensjonen. På tross av at CFIR omhandler hva som påvirker implementeringen, mens IOF handler om konsekvensene av implementeringen, inkluderer begge rammeverkene personers vurderinger av intervensjonen. Fremtidige implementeringsstudier bør ivareta transaksjonelle perspektiver.

## #7 Bærekraftig implementering av høyintensiv gangtrening for pasienter til rehabilitering etter hjerneslag

### J.M. Halvorsen, C. Henderson, M.G. Hågå, K. Bergseth, M. Byhring, T.L.B. Eggen, H. Gustavsen, I. Rosseland, J.E. Nordvik, T. G. Hornby & J.L. Moore, Oslo kommune, Forsterket Rehabilitering, Aker

**Innledning:** I FIRST-Oslo prosjektet ble høyintensiv gangtrening (HIGT) implementert i klinisk praksis ved to rehabiliteringsavdelinger i Oslo gjennom bruk av rammeverket Knowledge-to-Action (KTA). Implementeringen resulterte i betydelig forbedring på relevante utfallsmål, og intervensjonen ble innført som standard behandling ved rehabiliteringsavdelingene. Etter vellykket implementering er videre monitorering en viktig del av prosessen, og utgjør den siste fasen i KTA modellen. Monitoreringen gjøres for å kunne vurdere om ny praksis opprettholdes over tid. Presentasjonen tar for seg strategier brukt for å sikre bærekraftig implementering av HIGT, samt resultatene av en bærekraftighetsanalyse utført to år etter opprinnelig implementering.

**Metodebeskrivelse:** Presentasjonen viser strategier benyttet for å fremme bærekraftig implementering, med utgangspunkt i de tre domenene i «NHS Sustainability Model». Modellen brukes også til å evaluere strategiene og for å kartlegge forbedringsområder. Deretter tar presentasjonen for seg en analyse av bærekraftigheten til den opprinnelige implementeringen av HIGT. Bærekraftig praksis ble definert som opprettholdelse av gangrelatert trening og skrittaktivitet, høy kardiovaskulær intensitet, samt tilsvarende forbedring av funksjonelle utfallsmål.

**Resultater:** Tretten ulike strategier ble benyttet for å fremme bærekraftig implementering. Disse presenteres ut i fra respektive domener i «NHS Sustainability Model». Bærekraftighetsanalysen viste en lavere skrittaktivitet to år etter implementeringen, dog var forskjellen ikke statistisk signifikant. Kardiovaskulær intensitet i behandlingen og forbedring av funksjonelle utfallsmål var uendret.

**Diskusjon og konklusjon**: Resultatene av bærekraftighetsanalysen indikerte opprettholdt mengde gangrelatert trening, skrittaktivitet og kardiovaskulær intensitet i behandlingen to år etter implementeringen av HIGT. Behandlingen medførte også tilsvarende forbedring av funksjonelle utfallsmål, og således samme nytteverdi for pasientene ved avdelingen.

## #8 Utvikling og resultater av en vellykket implementeringsplan for høyintensiv gangtrening for pasienter etter hjerneslag

### Elisabeth Bø, Jennifer L. Moore, Anne Erichsen, Ingvild Rosseland, Joakim Halvorsen, Hanne Bratlie, T. George Hornby & Jan Egil Nordvik, Enhet for rehabilitering, OUS

**Innledning**: Høyintensiv gangtrening (HIGT) er en anbefalt intervensjon for personer med nedsatt gangfunksjon etter hjerneslag. Forskning indikerer dog at effektiv implementering av intervensjoner i klinisk praksis er en utfordring.

**Metode:** To rehabiliteringsenheter samarbeidet med et kunnskapstranslasjonssenter for å implementere HIGT i klinisk praksis. Data ble samlet inn under konvensjonell rehabilitering og etter implementering av HIGT. Vi utviklet en implementeringsplan ved hjelp av The Knowledge-to-Action cycle (KTA). Consolidated Framework for Implementation Research ble brukt til å identifisere barrierer og velge implementeringsstrategier. Ved å bruke et mixed-methods design med spørreundersøkelser, uformelle diskusjoner og dokumentasjon av praksis evaluerte vi tidligere og nåværende praksis, barrierer, resultater og opprettholdelse av HIGT over tid.

**Resultater:** En fler-komponents implementeringsplan med 26 implementeringsstrategier ble utviklet. Konvensjonelle tiltak for å forbedre gange inkluderte en kombinasjon av balanse, styrketrening og gangintervensjoner. Barrierer for bruk av HIGT inkluderte manglende kunnskap om intervensjonen, antakelser om ny praksis, evne til å tilpasse seg HIGT, ressurser og kultur. Spørreundersøkelser og uformelle diskusjoner identifiserte betydelige endringer i opplevd praksis og bruk av HIGT som følge av implementeringsplanen. Resultatene viste signifikant forbedring på gangrelatert utfallsmål og positive effekter på helsesystemet. Oppfølgingsundersøkelsen etter to år bekreftet at HIGT fortsatt ble gjennomført i praksis.

**Diskusjon og konklusjon:** Flere momenter var viktig for det gode resultatet; utvikling av en fler-komponents implementeringsplan, det var lettere enn forventet å fjerne eksisterende praksis, terapeutene og organisasjonen viste evne til og var klar for endringene som krevdes. Bruk av implementeringsrammeverk og en flere-komponent implementeringsstrategi kan øke effektiviteten i kliniske implementeringsprosjekter.

## #9 Undersøkelse av Ledelse og organisatorisk endring for implementering (LOCI) som en strategi for implementering av kunnskapsbasert praksis i psykisk helsetjeneste.

### Karina Egeland & Ane-Marthe Solheim Skar, Nasjonalt kunnskapssenter om vold og traumatisk stress

**Bakgrunn:** Til tross for at god ledelse ofte nevnes som en nødvendighet for å lykkes med implementeringen av kunnskapsbaserte behandlingsmetoder i helsetjenestene, er det lite forskning på hva god ledelse for implementering innebærer.

**Metodikk**: I denne studien ble det gjennomført en trinnvis klyngerandomisert kontrollert studie for å undersøke effektiviteten av en implementeringsstrategi kalt Ledelse og organisatorisk endring for implementering (LOCI). Totalt 47 psykiske helsetjenester for voksne og barn deltok. Klinikkledere og terapeuter fylte ut spørreskjemaer som vurderte ledelse og implementeringsklima ved oppstart og 4, 8, 12 og 16 måneder. Det ble antatt at LOCI ville være relatert til høyere terapeutvurdert implementeringsledelse, transformasjonsledelse og implementeringsklima når klinikken deltok i LOCI sammenlignet med perioden før LOCI-deltakelse.

**Resultater:** Resultatene bekreftet vår hypotese ved å demonstrere en signifikant økning i terapeutvurdert implementerings-, transformasjonsledelse og implementeringsklima etter klinikkenes oppstart i LOCI. Dette ble opprettholdt på alle målingstidspunktene. Før klinikkene deltok i LOCI var det en jevn nedgang i skårene.

**Diskusjon:** Dette er den første studien som tester LOCI-strategien i en ikke-amerikansk kontekst. Studien fremmer feltet ved å demonstrere effekten av LOCI-strategien på nøkkelfaktorer som er trukket fram som viktige for vellykket implementering av kunnskapsbasert praksis.

## #10 Systematisk oversikt over kunnskapstranslasjon i rehabiliteringsforskning.

### Julia Aneth Mbalilaki, Jenni Moore & Ian Graham, RKR, Sunnaas sykehus

**Innledning:** Knowledge-to-Action rammeverket (kunnskap til handlingsmodellen) er en systematisk kunnskapstranslasjons-metode for implementering av kunnskapsbasert praksis, med mål om å redusere gapet mellom forskning og praksis, ved hjelp av en trinnvis prosess med 7 faser.

**Hensikt:** Få oversikt over hvordan publiserte studier som har anvendt KTA rammeverket beskriver de ulike implementeringsfasene; identifisere aktiviteter utført i hver fase av KTA; og foreslå hvordan slike prosesser i rehabilitering kan forbedres.

**Metodebeskrivelse:** Litteratursøk i elektroniske databaser Google Scholar og PubMed, ble gjennomført frem til 31. desember 2019.

**Sammendrag av resultatene:** Totalt 49 artikler som rapporterte bruk av KTA i kliniske implementeringsprosjekter i rehabilitering ble inkludert i oppsummeringen.

**Diskusjon og konklusjon:** Oppsummeringen viser at implementering av kunnskapsbasert praksis ikke automatisk skjer som følge av ny evidens, men at det kreves målbevisst planlegging med stor innsats for å implementere og endre. Flere retningslinjer for kliniske spørsmål bør produseres, gjøres tilgjengelige og implementering bør følges av tett veiledning. Å inkludere all «stakeholders» fra klinikk og forskning når retningslinjer for klinisk praksis skal publiseres og lanseres. Viktig å se også hvordan intervensjon og evidens kan tilpasses slik at de er gjennomførbare i den lokale konteksten uten at man mister innhold og anbefaling. Potensielle barrierer for implementering på alle nivå, må avdekkes og løses på en systematisk måte. Godt samarbeid i forskjellige grupper av stakeholdere er også nevnt som en viktig nøkkel for å lykkes med en implementering. Bruk av standardiserte måleverketøy er også løftet frem som avgjørende faktorer for å løfte kvaliteten på implementering og vurdering.

## #11 Gjennomføring av en RCT i spesialisthelsetjenesten: Utfordringer og muligheter

### Trude Fredriksen, BUP Lillehammer/Otta SIHF/UIO

**Bakgrunn:** Det er behov for å etablere et evidensbasert tiltak for søsken som pårørende ved ulike hjelpeinstanser i Norge. Per i dag er det ingen systematiske og evidensbaserte tilbud til denne gruppen. SIBS-RCT er en randomisert kontrollert studie som har som primært mål å måle effekten av det kunnskapsbaserte gruppetiltaket SIBS på søskens psykiske helse. Hensikt med denne presentasjonen er å beskrive aktuelle utfordringer og muligheter erfart etter iverksetting av en RCT i spesialisthelsetjenesten.

**Metode:** Deltakere rekrutteres gjennom BUP og Habiliteringstjenesten for barn via behandlere de har kontakt med og informasjonshefter. Intervensjonen drives i ordinær arbeidstid og går over to halve arbeidsdager. Gruppeledere er behandlere ved poliklinikken som har fått opplæring i SIBS.

**Resultat/Erfaringer:** Datainnsamlingen startet i mai 2019 og 50 grupper er gjennomført med totalt 170 søsken. Utfordringer er tilgang på nødvendige ressurser som personell, tid, romfasiliteter, datautstyr og kontormateriell. Behandlere glemmer å tilby tiltaket. Vanskelig å rekruttere deltakere. Ofte er det behov for ekstra kontakt og informasjon før deltakere samtykker. Deltakere har manglende forståelse av tiltaket til tross for å ha mottatt mye informasjon. Gode tilbakemeldinger fra deltakere som har gjennomført gruppetiltaket.

**Konklusjon:** Nødvendige forutsetninger for å gjennomføre en randomisert kontrollert studie av et gruppetiltak i spesialisthelsetjenesten, er lederforankring på flere nivå, stedlig ansvarlig person på enheten, hyppig informasjon om og påminning av tiltaket i kollegagruppen.

## #12 Implementering av en integrert kunnskapsoversettelsesintervensjon i sykehjem: erfaringer fra praksisutviklingssykepleiere (practice development nurses)

### Trine Lise Steinskog, O. Tranvåg, M. Nortvedt, D. Ciliska & B. Graverholt, Høgskulen på Vestlandet

**Bakgrunn:** Praksisutviklingssykepleiere (practice development nurses; PDN) i norske sykehjem har et spesifikt ansvar for å overføre forskning til en stadig mer kompleks praksis. PDN-er er involvert som sluttbrukere i IMPAKT-intervensjonen (IMPlementation of Action to Knowledge Translation). Ved å bruke en integrert kunnskapsoversettelsestilnærming, deltok de i et utdanningsprogram skreddersydd for deres identifiserte behov. I en andre komponent brukte de læringen sin i tilrettelegging ved implementering av verktøyet National Early Warning Score (NEWS2). Målet med denne studien var å utforske PDNs erfaringer med å delta i en IKT-pedagogisk intervensjon, og hvordan de brukte læringen i planlegging, skreddersying og innledende implementering av NEWS2.

**Metode**: En kvalitativ eksplorativ studie basert på en fenomenologisk hermeneutisk metode. Vi gjennomførte ni dybdeintervjuer av PDN-ene og åtte ikke-deltakende observasjonssesjoner av implementeringsstrategien/leveransen.

**Resultater**: PDN-ene ga uttrykk for at utdanningsprogrammet dekket deres behov og økte deres forståelse av ledende kunnskapsoversettelse (KT). De rapporterte en overgang fra å operere i en "stor svart boks for implementering" til et profesjonelt og strukturert modus for KT. PDN-ene rapporterte økt kompetanse i KT og i deres evne til å involvere og samarbeide med andre i deres anlegg. Organisatoriske kontekstuelle faktorer utfordret deres KT-innsats og implementering av NEWS2.

**Konklusjon:** Denne studien viser at en IKT-tilnærming har potensialet til å fremme og forbedre personalkompetanser og NH-beredskap for KT. Individuell motivasjon og kompetanse ble imidlertid utfordret innenfor en organisasjonskultur som var mindre mottakelig for denne nye lederrollen og nivået av KT-aktivitet.

## #13 Valideringer av Implementation Leadership Scale, Implementation Climate Scale, Implementation Citizenship Behavior Scale og AIM-, IAM- og FIM-skalaene i en norsk setting for psykisk helsevern

### Nora Braathu, Randi Hovden Borge, Mathilde Endsjø & Nadina Peters, Nasjonalt kunnskapssenter om vold og traumatisk stress (NKVTS)

**Bakgrunn:** Implementering av evidensbasert praksis (EBP) er av avgjørende betydning i helseinstitusjoner. Vellykket implementering kan påvirkes av implementeringsledelse, implementeringsklima, implementeringsborgerskapsatferd (ansattes ekstrarolleatferd for å støtte EBP-implementeringen), og oppfatninger av EBP. Den nåværende studien kombinerer fire psykometriske studier på instrumenter som brukes til å måle disse konstruksjonene i sammenheng med EBP-implementering. Dette var første gang disse instrumentene ble validert i norsk sammenheng.

**Mål:** Målet med de nåværende studiene var å vurdere de psykometriske egenskapene til seks instrumenter som ble brukt ved implementering av EBP i et norsk helsevesen.

**Metode:** Følgende skalaer ble brukt: The Implementation Leadership Scale (ILS), The Implementation Climate Scale (ICS), The Implementation Citizenship Behavior Scale (ICBS) og Feasibility of Intervention Measure (FIM), Intervention Appropriateness Measure (IAM) og Acceptability of Intervention Measure (AIM). Alle instrumenter ble utsatt for bekreftende faktoranalyse.

**Resultater**: Alle kartleggingsskjemaene viste aksepterte psykometriske egenskaper.

**Konklusjoner**: Resultatene tyder på at ILS, ICS, ICBS og FIM, IAM, AIM er valide og pålitelige verktøy for å måle disse implementeringskonseptene i det norske helsevesenet.

## #14 Kort innføring i KTA Knowledge to Action rammeverket

### Stein Arne Rimehaug, RKR, Sunnaas sykehus

Knowledge-to-Action (KTA) rammeverket er ment å favne hele evidensgrunnlaget fra implementeringsvitenskap, og være en hjelp i å sikre en mer helhetlig kunnskapstranslasjonsprosess. Flere andre presentasjoner som er submittert for NIMP 2021 konferansen omhandler avsluttede prosjekter som har brukt dette rammeverket, så det vil være nyttig å også få med en kort presentasjon av selve rammeverket, visualisert i KTA modellen fraStraus, Tetroe & Graham sin bok fra 2013 «Knowledge Translation in Health Care: Moving from Evidence to Practice». Et lite antall andre implementeringsprosjekter i norsk helsetjeneste har blitt inspirert av en animert Youtube instruksjonsvideo om KTA rammeverket «Fra kunnskap til handling» fra RKR, med til nå 2600 visninger, som også er lagt ut å Kunnskapssenterets nettressurs om kunnskapstranslasjon. Denne animasjonsvideoen ble laget i 2014 av Stein Arne Rimehaug, som også vil presentere KTA rammeverket i en 10-15 minutter lang presentasjon, sammen med Julia Mbalilaki, også fra RKR, Regional Kompetansetjeneste for Rehabilitering. Sekundært kan dette presenteres som en poster, men det vil bli et langt bedre læringsutbytte og helhet av å kunne presentere dette i plenum i forbindelse med andre presentasjoner som omhandler KTA.

## #15 Søsken som pårørende: En kartlegging av forebyggende psykisk helsearbeid i norske kommuner og prospektiv aksept av det gruppebaserte tiltaket SIBS

### Yngvild B. Haukeland, Torun M. Vatne, Ann-Helen Kongshavn & Ragnhild Bang Nes, Psykologisk institutt, Universitetet i Oslo

**Bakgrunn:** Helsepersonell er pliktet til å ivareta søsken som pårørende, men vi vet lite om etterlevelse av dette i norske kommuner. SIBS er en kunnskapsbasert søskenintervensjon med formål å forebygge psykisk uhelse. For vellykket spredning av SIBS trengs kunnskap om kommuneansattes vurderinger av tiltaket og antatte implementeringshindre.

**Metode:** Spørreskjema ble høsten 2019 sendt til helsesykepleiere, kommunepsykologer og kommuneoverleger for å kartlegge identifisering av søsken, samarbeid med spesialisthelsetjenesten og eksisterende tilbud til søsken. Vi kartla videre prospektiv aksept av SIBS og analyserte åpne utsagn om tiltakets styrker og svakheter med en deduktiv innholdsanalyse ut fra Consolidated Framework for Implementation Research.

**Resultater:** 332 informanter (58,9% helsesykepleiere) fra 253 kommuner deltok. Identifisering av søsken ble rapportert å hovedsakelig skje tilfeldig (62,1%) og sjeldent systematisk (11,5%). Majoriteten (66%) rapporterte ingen eller lite samarbeid med spesialisthelsetjenesten om pårørende søsken. Tilbud ble oftest gitt av skolehelsetjenesten (83,7%). Individuell samtale etter forespørsel fra familien var vanligst (88,9%) og gruppesamtaler sjeldent (11,1%). SIBS’ kvalitet ble vurdert som god og gjennomførbarhet som moderat. Vi fant at oppfattede styrker hovedsakelig omhandlet tiltakets kjennetegn (f.eks. gjennomtenkt og systematisk), samt individkarakteristikker (f.eks. opplevd samsvar med egne verdier og erfaringsgrunnlag). Oppfattede svakheter omhandlet hovedsakelig kontekstuelle faktorer; særlig begrenset tilgang på deltakere og fagpersoner i små kommuner og manglende samarbeid på tvers av kommuner og tjenestenivå.

**Konklusjon**: Videre arbeid med pårørende søsken bør fokusere på samarbeid mellom kommune og spesialisthelsetjenesten for å sikre at søsken identifiseres. Interkommunalt samarbeid bør også tilstrebes for å sikre tilstrekkelige ressurser til at kunnskapsbaserte intervensjoner som SIBS kan implementeres.

## #16 En veileder for kunnskapstranslasjon og implementering – på norsk!

### Birgitte Graverholt, Hilde Strømme & Donna Ciliska, Høgskulen på Vestlandet

**Bakgrunn:** Kunnskapsbasert praksis har et godt fotfeste i Norge og internasjonalt og diskusjoner handler ikke lenger om man skal jobbe kunnskapsbasert, men hvordan få det til. Opplæring i kunnskapsbasert praksis har pågått i mange år, men denne har i mindre grad hatt fokus på de trinnene som handler om å implementere og evaluere praksis. Registered Nurses of Ontario (RNAO) har utviklet en omfattende og kunnskapsbasert veileder for implementering av anbefalinger fra forskning. Veilederen kan brukes for å informere implementering, og som grunnlag for undervisning og opplæring i implementering.

**Hensikt:** Å oversette en veileder for implementering av kunnskapsbaserte anbefalinger, til norsk.

**Metode:** Oversettelsen av «Toolkit: Implementation of Best Practice Guidelines” skjedde i tre trinn: Et profesjonelt oversettelsesbyrå gjorde den første oversettelsen. En person med ekspertise i kunnskapsbasert praksis gjennomgikk oversettelsen og sikret tilpassing til norsk og faglig kontekst. Til sist leste en sykepleier hele veilederen for å sikre relevans for klinisk arbeid.

**Resultat:** Veilederen «Verktøykasse: Implementering av kunnskapsbaserte retningslinjer» finnes nå på norsk og er tilgjengelig for alle i Norge, gjennom lenke fra www.kunnskapsbasertpraksis.no (Helsebiblioteket). Veilederen er bygget opp rundt kunnskap-til-handling rammeverket. Denne modellen har blitt brukt i et stort implementeringsprosjekt (IMPAKT) og også systematisk i utvikling av et 15 studiepoengs emne om implementering på Master i kunnskapsbasert praksis i helsefag, HVL.

**Konklusjon:** Kunnskapsbasert praksis er bare staffasje om det ikke integreres i klinisk praksis. Helsepersonell har lenge manglet gode ressurser som kan støtte implementering av ny kunnskap, mens undervisere i kunnskapsbasert praksis har undervurdert kompleksiteten av å implementere. Denne omfattende veilederen kan brukes i begge sammenhenger, enten i sin helhet, eller kapittelvis og bidra til at pasienter møter en kunnskapsbasert helsetjeneste.

## #17 IPIC-studien: Effekten av et tverrprofesjonelt læringsprogram for brukermedvirkning blant eldre personer i korttidsrehabilitering: En kvasi-eksperimentell studie

### Linda Aimée Hartford Kvæl, Oslo Metropolitan University

**Bakgrunn:** Kommunal korttidsrehabilitering (KKR) fungerer som en bro mellom sykehus og hjem for eldre personer med kompleks problematikk. Brukermedvirkning betyr at de som mottar hjelp har rett til å medvirke. På tross av at brukermedvirkning er en demokratisk rettighet og et politisk mål, viser forskning at eldre pasienter og deres pårørende i KKR ikke opplever tilstrekkelig involvering. Helsepersonell rapporterer likeledes at brukermedvirkning er vanskelig å få til i den kliniske hverdagen. Det er således behov for økt kunnskap om hvordan implementere brukermedvirkning i KKR.

**Metode:** Gjennom IPIC-studien er målet å utvikle og evaluere en læringsintervensjon for helsepersonell i KKR basert på tverrfaglig simuleringsmetodikk. Undervisningsopplegget vil ta utgangspunkt i evidensbaserte kritiske punkter for brukermedvirkning i denne konteksten: 1) innkomsten 2) den tverrfaglige oppstartssamtalen og kontekstualisering av "Hva er viktig for deg?" 3) samarbeid bydel-helsehus 4) gode måltidsopplevelser 5) et rehabiliterende miljø og 6) utskrivelsen til hjemmet. Som del av undervisningsopplegget vil vi utvikle en kortfilm basert på real-life scenarioer som setter søkelyset på hvordan brukermedvirkning faktisk kan fremmes i den kliniske hverdagen. Læringsintervensjonen skal utvikles i samarbeid med praksisfeltet og gjennomføres i én korttidsavdeling gjennom tre halvdagsseminarer og fire oppfølgingssamtaler og sammenlignes med en annen tilsvarende avdeling som kontroll.

**Resultater:** I tillegg til å prosessevaluere læringsintervensjonen bant deltakerne vil vi vurdere effekten på brukermedvirkning, fysisk funksjon, pasienttilfredshet og pårørendebelastning. Økt kunnskap og bevissthet hos helsepersonell er en viktig implementeringsstrategi. Brukermedvirkning er assosiert med bedre utfall av rehabiliteringen, økt pasienttilfredshet, styrket autonomi og livskvalitet. Målet er økt implementering av brukermedvirkning i KKR.

## #18 Oversettelse og validering av Alberta Context Tool for bruk i norske sykehjem

### Jannicke Igland, Thomas Potrebny, Bente E. Bendixen, Anne Haugstvedt, Birgitte Espehaug, Kristine B. Titlestad & Birgitte Graverholt, Høgskulen på Vestlandet

**Bakgrunn:** Organisatorisk kontekst er anerkjent som viktig for å tilrettelegge for evidensbasert praksis og forbedre pasientresultater. Organisatorisk kontekst er et komplekst konstrukt å måle, og passende instrumenter som kan kvantifisere og måle kontekst er nødvendig.

**Hensikt:** Målet med denne studien var å oversette og tverrkulturelt tilpasse Alberta Context Tool (ACT) til norsk, og å teste reliabiliteten og strukturell validitet blant registrerte sykepleiere (RNer) og lisensierte praksissykepleiere (LPNs) som arbeider i sykehjem.

**Metoder:** Denne studien var en valideringsstudie som benyttet et tverrsnittsdesign. Utvalget besto av n = 956 helsepersonell fra 28 sykehjem fra en kommune i Norge. I den første fasen ble ACT oversatt før den ble administrert på 28 sykehjem. I den andre fasen ble intern konsistens og strukturell validitet utforsket ved hjelp av Cronbach’s alfa- og bekreftende faktoranalyse.

**Resultater:** En streng frem-og-bakoversettelsesprosess ble utført, utført av et team av akademikere, eksperter, profesjonelle oversettere og rettighetshaverne, før en akseptabel versjon av ACT ble testet og ferdigstilt. Den norske versjonen av ACT viste god intern konsistens med Cronbach’s alfa over .75 for alle konsepter bortsett fra Formal interactions hvor alfa var .69. Strukturell validitet var akseptabel for både RN-er og LPN-er med faktorladninger over 0,4 for de fleste varer.

**Konklusjoner:** Den norske versjonen av ACT er et gyldig mål på organisatorisk kontekst i norske sykehjem blant RNer og LPNer. Den norske versjonen av ACT kan derfor tjene som et viktig verktøy i fremtidige implementeringsstrategier og forskningsprosjekter.
